# Epigenetic remodeling in sarcoma promotes T-cell infiltration via modulation of the Hippo pathway

**DOI:** 10.1136/jitc-2025-014601

**Published:** 2026-04-01

**Authors:** Mireia Cruz De los Santos, Yi Chen, Amaia González De Zárate, Agnes Sorteberg, Honglei Zhao, Guillermo Vázquez-Cabrera, Neda Bigdeli, Solrun Kolbeinsdottir, Aarren Mannion, Lucas Baldran-Groves, Shi Yong Neo, Stina Linnea Wickström, Jeroen Melief, Lars Holmgren, Nikolas Herold, Felix Haglund de Flon, Andreas Lundqvist

**Affiliations:** 1 Department of Oncology-Pathology, Karolinska Institutet, Stockholm, Sweden; 2 Division of Hematology and Oncology, Columbia University, New York, New York, USA; 3 Department of Women's, and Children's Health,Childhood Cancer Research Unit, Karolinska Institutet, Solna, Sweden; 4 Institute of Environmental Medicine, Toxicology Unit, Karolinska Institutet, Solna, Sweden; 5 Singapore Immunology Network, Agency for Science, Technology and Research, A*STAR, Singapore; 6 Section Pediatric Oncology, Astrid Lindgren Children’s Hospital, Stockholm, Sweden; 7 Department of Pathology and Cancer Diagnostics, Karolinska University Hospital, Stockholm, Sweden

**Keywords:** Sarcoma, Immunotherapy, Memory, T cell, Tumor infiltrating lymphocyte - TIL

## Abstract

**Background:**

Insufficient T-cell infiltration limits the effectiveness of immunotherapy in sarcoma, yet the tumor-intrinsic mechanisms that govern immune exclusion remain poorly defined.

**Methods:**

By integrating patient-derived ex vivo sarcoma spheroids with autologous expanded tumor-infiltrating lymphocytes and an in vivo metastatic osteosarcoma model, antitumor immune regulation by histone modifications was examined.

**Results:**

Histone H3 lysine 27 acetylation (H3K27ac) was identified as a key regulator of CD8^+^ T-cell infiltration in osteosarcoma and other bone and soft-tissue sarcomas. Pharmacological elevation of H3K27ac by the histone deacetylase 1/3 inhibitor entinostat promotes CD8^+^ T-cell activation, cytotoxicity, and the recruitment of CD8^+^CD103^+^ tissue-resident memory T cells. Mechanistically, these immune-boosting effects are triggered by a Hippo pathway switch, in which yes-associated protein 1 (YAP1) is suppressed, and vestigial-like family member 3 (VGLL3) is induced, thereby modulating transcription towards an immune-responsive state. Furthermore, we identified that VGLL3/CD103 signatures predict a response to anti-programmed cell death protein-1 (PD-1) treatment in patients with sarcoma, and that combining H3K27ac induction with anti-PD-1 further augments T cell-mediated killing in ex vivo autologous patient-derived spheroid models.

**Conclusions:**

Our findings reveal an epigenetic-Hippo-immunomodulatory axis in osteosarcoma that also extends to other sarcomas, providing a rationale for incorporating epigenetic preconditioning with immunotherapy to improve patient outcomes and pointing towards novel biomarkers for treatment guidance.

**WHAT IS ALREADY KNOWN ON THIS TOPIC**

- Sarcomas exhibit poor T-cell infiltration, which limits the effectiveness of immune checkpoint therapy.

**WHAT THIS STUDY ADDS**

- Using patient-derived and murine sarcoma models, we show that epigenetic reprogramming to increase tumor H3K27ac modulates the Hippo pathway to favor CD8^+^ T-cell activation and infiltration of tissue-resident T cells.

**HOW THIS STUDY MIGHT AFFECT RESEARCH, PRACTICE OR POLICY**

- The Hippo pathway factors yes-associated protein 1 (YAP1) and vestigial-like family member 3 (VGLL3) represent promising targets to develop novel cancer immunotherapies and predictive biomarkers for immune checkpoint therapy.

## Background

 Sarcomas are heterogeneous mesenchymal tumors classified as soft-tissue sarcoma (STS) and bone sarcomas, the latter including osteosarcoma (OS). While STS accounts for 1% of adult cancers, OS accounts for nearly 4% of pediatric malignancies, representing 20% of bone cancers.^1^,^3^ The treatment strategies for sarcomas have not evolved at the same pace as for many other cancer types, resulting in a plateau in survival outcomes over the past 40 years.^4^ With a high recurrence rate and patients presenting with micrometastasis already at diagnosis,^5^ the survival rate of unresectable OS remains below 15%,^6^ highlighting the need for improved treatment strategies.

While cancer immunotherapy has revolutionized the clinical outcome for several cancers, treatment with immune checkpoint inhibitors (ICI) has largely failed in OS and STS. This is attributed to an immunosuppressive tumor microenvironment (TME) characterized by limited CD8^+^ T-cell infiltration.^7 8^ In contrast, sarcomas with a more inflamed TME, such as undifferentiated pleomorphic sarcoma, respond better to ICI.^8^ These observations underscore the need to identify biomarkers of response and develop strategies to reshape the TME from being immunosuppressive to inflamed.

Epigenetic dysregulation is increasingly recognized as a major driver of sarcomagenesis.^9^ Among the various epigenetic mechanisms, histone modifications occurring on the N-terminal residues of histone tails, which modulate chromatin accessibility, have received limited attention in OS and STS. In fact, genome-wide patterns of histone modifications in tumor cells are notably altered in metastatic OS lesions compared with localized lesions.^10^ Yet, how tumor chromatin states shape the immune landscape remains poorly understood. Here, we investigate whether specific histone modifications can reprogram the immune microenvironment of sarcoma and found that elevated levels of histone 3 lysine 27 acetylation (H3K27ac) in tumor cells correlate with increased CD8^+^ T-cell infiltration. Using unique ex vivo patient-derived spheroid models along with expanded autologous T cells and metastatic OS in vivo models, we identified that pharmacological enhancement of H3K27ac using the histone deacetylase inhibitor, entinostat, modulates the Hippo pathway transcriptional enhanced associate domain (TEAD) cofactors, yes-associated protein 1 (YAP1), and vestigial-like family member 3 (VGLL3), resulting in increased tumor infiltration by tissue-resident memory CD8^+^ T cells and enhanced cytotoxic function. Our results identify H3K27ac as a putative biomarker, and that modulation of the Hippo pathway can augment T-cell infiltration and synergize with immunotherapies in patients with sarcoma.

## Methods

### Cell culture

Patient-derived cells were isolated from surgically resected sarcoma samples obtained under ethical permits 2013/1979-31 and 2022–05409-01. Healthy donor buffy coats were obtained under ethical permit 2024–07830-01. After peripheral blood mononuclear cell (PBMC) isolation from healthy donors using Ficoll Paque (Cytiva) T cells and monocytes were isolated by the Human Pan T cell Isolation Kit (Miltenyi, 130–096-535) and CD14 Human MicroBeads (Miltenyi, 130–050-201), respectively. Whole PBMC and isolated T cells were cultured in tumor-infiltrating lymphocyte (TIL) medium for 24 hours prior to co-culture with tumor cells. CD14^+^ isolated cells were cultured in Roswell Park Memorial Institute medium (RPMI) 1640 (Gibco, 11875093), 5% fetal bovine serum (FBS), and 1% penicillin/streptomycin. Refer to online supplemental methods for detailed information.

The sarcoma commercial cell lines U2OS, SAOS2, and SJSA1 were obtained from American Type Culture Collection (ATCC). U2OS and SAOS2 were cultured in McCoy medium supplemented with 10% FBS and 1% penicillin-streptomycin. SJSA1 cells were cultured in RPMI 1640 supplemented with 10% FBS and 1% penicillin-streptomycin. K7M2 murine OS cells were cultured in Dulbecco’s Modified Eagle Medium (DMEM) GlutaMAX supplemented with 10% FBS, 1% penicillin-streptomycin, and 1% pyruvate.

For spheroid cultures, cells were resuspended in tumor spheroid culture medium (DMEM/F-12 (1:1) (1x) (Gibco, 31330–038) supplemented with 1% penicillin-streptomycin, 1x GlutaMAX (Gibco, 35050–061), and 20% heat-inactivated FBS). Between 5,000 and 10,000 cells per well were seeded in Nunclon Sphera 96-well U-bottom plate (Thermo Fisher Scientific, 174929). Thereafter, 100 µL per well of cold spheroid culture medium containing 4% Geltrex (Gibco, A14132-02) was added to patient-derived cultures. Cells were cultured for 3–5 days to allow spheroid formation. Autologous TIL or allogenic PBMCs or T cells were resuspended in spheroid medium and added to tumor spheroids at a 1:1 to 10:1 effector:target ratio. Where indicated, cells were treated with entinostat (Selleckchem, S1053) at indicated concentrations or 0.1 µg/mL verteporfin (MedChemExpress, HY-B0146R). TILs were pretreated with nivolumab at indicated concentrations 30 min before co-culture. Subsequent analyses are described in online supplemental methods.

### Real-time killing assay

To assess TIL-mediated killing, Green CellEvent Caspase-3/7 Detection Reagent (Thermo Fisher Scientific, C10723) was added to a final concentration of 1:5,000 prior to co-culture of spheroids with TILs. Co-cultures were imaged using IncuCyte S3 ESSEN (Bioscience). Brightfield and green channel images were acquired every hour at 4× magnification. Incucyte Software (V.2024A, Sartorius) was used for analysis as described in online supplemental methods.

### Lymphocyte infiltration analysis

Tumor infiltration of lymphocytes was assessed by three-dimesional confocal imaging as previously described.^11^ For infiltration analysis by flow cytometry, tumor spheroids were washed three times with phosphate-buffered saline (PBS) to eliminate non-infiltrating cells. At least six spheroids per condition were transferred to a 96-well V-bottom plate for disaggregation with TrypLE Express (Gibco, 12604–013). To stop the reaction, Fluorescence-activated cell sorting (FACS) buffer (PBS 5% FBS, 2 mM EDTA) was added until a homogeneous single-cell suspension was obtained. Cells were then acquired by flow cytometry as described in online supplemental methods.

### In vivo experiments

In vivo experiments were reviewed and approved by the Stockholm Regional Ethics Committee (13820-2019 and 4356–2024). BALB/C mice (females, 4–5 weeks) were purchased from Taconic Biosciences, USA. The experiment was repeated at two different time points, with five to six animals per group. On day 0, all mice were intravenously injected with 500,000 K7M2 cells. After allowing the establishment of lung micrometastases for 2 days, the chow was changed to entinostat diet (10 mg/kg mouse body weight, personalized and formulated by Inotiv-Teklad Diet) or a control diet (prepared according to entinostat chow at Inotiv-Teklad) until day 20. Mice had ad libitum food availability and water. All mice were monitored daily for signs of distress or pain, according to the Mouse Grimace Scale and the previously stated humane endpoints. On day 20, all mice were euthanized by CO_2_ followed by cervical dislocation. Tissues were processed for immunohistochemistry or into single-cell suspensions for flow cytometry analysis as described in online supplemental methods.

Additional materials and methods are provided in the online supplemental methods.

## Results

### H3K27ac levels correlate with survival and immune infiltration in sarcoma

**Figure 1:**
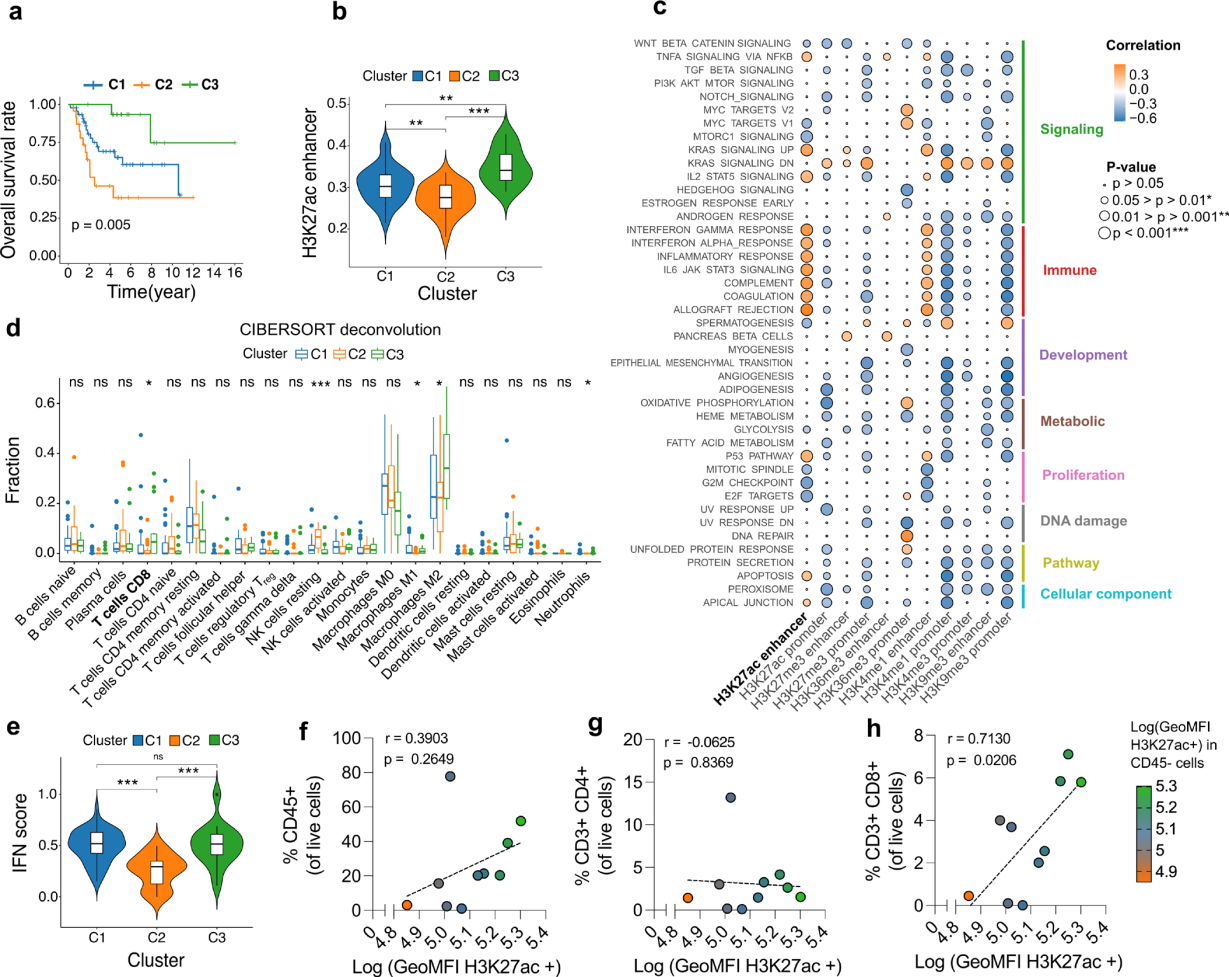
H3K27ac correlates with survival and immune signatures in sarcoma. (**a**) Overall survival of patients in clusters C1 (n=45), C2 (n=23), C3 (n=16) generated by unsupervised clustering of the TARGET cohort. Log-rank test was used to determine statistical significance. (**b**) Overall H3K27ac modification levels in enhancer regions in C1-C3. P values are calculated by one-way ANOVA. (**c**) Pathway enrichment analysis and correlation of each term with different histone modifications. (**d**) CIBERSORT deconvolution of immune fractions for patients in C1-C3. One-way ANOVA was used to calculate statistical significance for each population. (**e**) IFN score for patients in C1-C3. P value was calculated by one-way ANOVA. (**f–h**) Flow cytometry analysis for H3K27ac, CD45^+^, CD3^+^CD4^+^, and CD3^+^CD8^+ ^in freshly surgical resected samples from patients with soft tissue sarcoma, bone sarcoma, including patients with osteosarcoma (n=10). P values for (**f–g**) were calculated with Pearson correlation of cell frequencies with log transformed GeoMFI of H3K27ac in H3K27ac^+^ tumor cells (CD45^−^). *p<0.05, **p<0.01, and ***p<0.001. ANOVA, analysis of variance; IFN, interferon; ns, non-significant; TARGET, Therapeutically Applicable Research to Generate Effective Treatments.

To identify histone-modification patterns associated with clinical outcome, single-sample Gene-Set Enrichment Scores (ssGSEA) were computed from RNA sequencing (RNA-seq) expression profiles for histone-modification-defined gene sets (H3K27ac, H3K4me1/3, H3K36me3, H3K27me3, H3K9me3) at promoters and enhancers from patients of the Therapeutically Applicable Research to Generate Effective Treatments - Osteosarcoma (TARGET-OS) cohort.^12^ Principal component analysis of these epigenome scores revealed a clear separation between patients with OS (TARGET-OS) and healthy tissue controls from the Genotype-Tissue Expression (GTEx), indicating marked differences in their global transcriptional and epigenetic profiles (online supplemental figure 1a). Consistently, OS samples displayed higher enrichment scores than non-malignant controls for all histone modifications, thus confirming a globally altered chromatin landscape (online supplemental figure 1b). Next, unsupervised consensus clustering of epigenome scores identified three distinct clusters (C1-3) with significant differences in survival (figure 1a, online supplemental figure 1c). On assessment of histone modification levels across the three clusters, only the genome-wide levels H3K27ac at enhancer regions exhibited significant pairwise differences across all three clusters, which also corresponded with overall survival (figure 1b and online supplemental figure 1d).

To investigate why patients with H3K27ac-high tumors exhibited improved survival, pathway-level associations were examined. Hallmark analysis revealed a strong positive correlation between H3K27ac at enhancers and immune-related programs (figure 1c). Consistently, CIBERSORT deconvolution further indicated an enrichment for CD8^+^ T cells, neutrophils, M1/M2-like macrophages (figure 1d), and higher interferon scores in H3K27ac-high tumors (figure 1e). Application of ssGSEA H3K27ac scores to a heterogeneous database of STS tumors (The Cancer Genome Atlas – Sarcoma (TCGA-SARC))^12^ revealed concordant associations between elevated H3K27ac and increased CD8^+^ T cells (online supplemental figure 1e,f). Altogether, suggesting increased presence of cytotoxic T cells in H3K27ac-high OS and STS tumors (C3). To validate these findings, flow cytometry analysis of freshly resected OS and different STS tumors, representing multiple histologies, was performed. Indeed, assessment of these samples revealed that H3K27ac levels in tumor cells significantly correlated with CD8^+^ T-cell infiltration but not with CD4^+^ T-cell or total CD45^+^ immune cell infiltration (figure 1f–h, online supplemental methods figure 1, and online supplemental table 1). Together, these findings demonstrate that a permissive epigenetic state, as per H3K27ac levels, correlates with CD8^+^ T-cell infiltration and is associated with favorable patient outcomes, providing a rationale for epigenetic strategies that increase H3K27ac to remodel the TME.

### Pharmacologic increase of H3K27ac enhances T-cell infiltration in osteosarcoma

Since H3K27ac levels correlated with CD8^+^ T-cell frequency in tumors, we sought to investigate whether modulation of H3K27ac influenced T-cell infiltration in sarcoma. The class I histone deacetylases (HDAC) 1 and 3 are primary regulators of H3K27ac,^13^ and are involved in eliminating acetyl residues from H3K27. Notably, analysis of single-cell RNA-seq data from synovial sarcoma samples (GSE131309)^14^ revealed that HDAC1/3 are overexpressed in tumor cells compared with stroma or immune cells (online supplemental figure 1g). Analysis of HDAC1/3 expression across H3K27ac-defined clusters (C1–C3) in STS and OS cohorts showed higher HDAC1/3 levels in the H3K27ac-low cluster (C2), in line with reduced H3K27 acetylation in this group. Moreover, elevated HDAC1/3 levels were associated with poorer patient survival (online supplemental figure 1h,i).

Therefore, to assess whether pharmacological HDAC1/3 inhibition altered H3K27ac levels, the selective HDAC1/3 inhibitor entinostat was used in tumor spheroids derived from OS cell lines: U2OS, SAOS2, and SJSA1 (online supplemental figure 2a). Flow cytometry analysis revealed distinct H3K27ac populations, with U2OS exhibiting the highest baseline frequency of H3K27ac-bright cells. Entinostat treatment increased H3K27ac-bright populations in SJSA1 and SAOS2 spheroids and enhanced H3K27ac levels across all cell lines (online supplemental figure 2b–d).

To assess the effect of H3K27ac modulation on immune infiltration, spheroids were exposed to subapoptotic levels of entinostat (online supplemental figure 2e), resulting in significantly higher tumor infiltration by healthy donor PBMCs and T cells (online supplemental figure 2f–j). In addition, we found that baseline tumor H3K27ac levels correlated with T-cell infiltration, which was further enhanced by entinostat treatment (online supplemental figure 2k). Together, these findings demonstrate that pharmacological inhibition of HDAC1/3 increases H3K27ac levels and improves immune infiltration in OS spheroids.

### H3K27ac induction promotes infiltration of tissue-resident memory CD8^+^ T cells in patient-derived sarcoma spheroids

**Figure 2:**
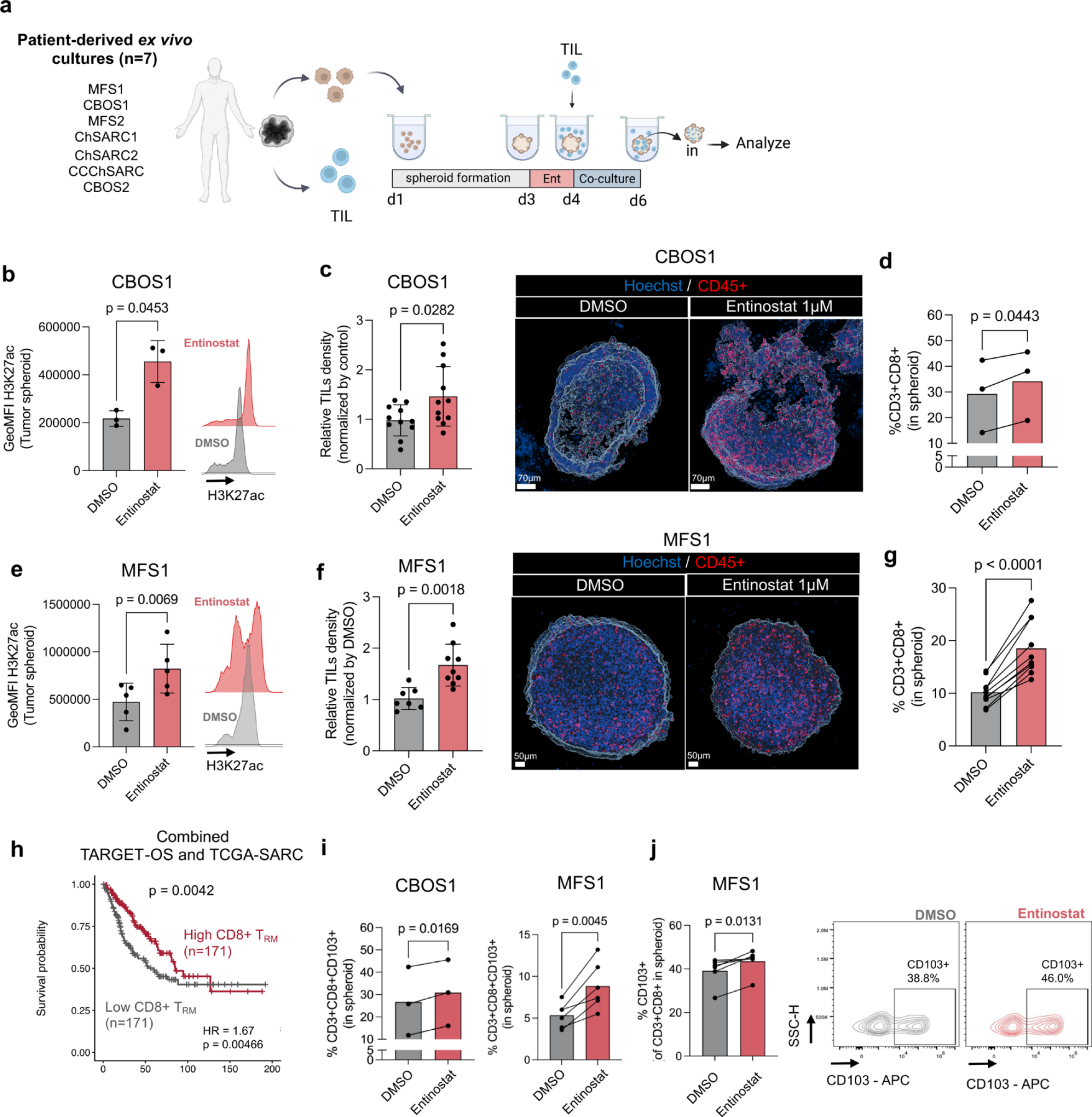
Entinostat increases H3K27ac in ex vivo patient-derived sarcoma spheroids and increases infiltration of autologous TILs and tissue-resident memory T cells. (**a**) Experimental design for T-cell infiltration assay. (**b**) H3K27ac GeoMFI of patient-derived CBOS1 cells after 24 hours of treatment with entinostat. (**c**) (Left) CBOS1-TIL density in spheroids by confocal microscopy relative to DMSO-treated controls. Each dot represents one spheroid culture, divided into three separate independent experiments. (Right) Representative confocal image of CBOS1 spheroids. (**d**) Flow cytometry analysis of CD8^+^ TIL infiltration in CBOS1 spheroids. (**e**) H3K27ac GeoMFI of patient-derived MFS1 cells after 24 hours of treatment with entinostat. (**f**) (Left) MFS1-TIL density in spheroids by confocal microscopy relative to DMSO-treated controls. Each dot represents one spheroid culture, divided into three separate independent experiments. (Right) Representative confocal image of MFS1 spheroids. (**g**) Flow cytometry analysis of CD8^+^ TIL infiltration in MFS1 spheroids. (**h**) TCGA sarcoma (TCGA-SARC) and TARGET osteosarcoma (TARGET-OS) Kaplan-Meier curves of patients divided by high (n=171) and low (n=171) CD8^+^ T_RM_ signatures, divided by median value. (**i**) (Left) Frequency of CD3^+^/CD8^+^/CD103^+^ T_RM_ cells within CBOS1 and (MFS1 spheroids, and frequency of CD103^+^ cells of infiltrated CD3^+^/CD8^+^ cells in (Right) MFS1 spheroids. (**j**) Population gating within the CD8^+^ T cells of CD103^+^ and representative gating of CD103^+^ cells in CD3^+^/CD8^+^ MFS1-TIL. APC, antigen presenting cell; DMSO, dimethyl sulfoxide; OS, osteosarcoma; TCGA-SARC, The Cancer Genome Atlas – Sarcoma; TARGET, Therapeutically Applicable Research to Generate effective treatments; T_RM_, tissue-resident memory.

To substantiate the association of H3K27ac and T-cell infiltration in a more relevant model, TILs were expanded from sarcoma patient resections and tested for their ability to infiltrate autologous ex vivo patient tumor spheroids (figure 2a, and online supplemental methods figure 2a–c). Indeed, entinostat increased H3K27ac levels and TIL infiltration in a chondroblastic OS patient-derived tumor spheroid (CBOS1). These results were reproduced in multiple patient-derived TIL-tumor autologous spheroid cultures, including one additional chondroblastic OS, two myxofibrosarcomas, and three chondrosarcoma cases, suggesting the extension of our findings to STS and other bone sarcomas (figure 2b–g, online supplemental figure 3a,b, and online supplemental table 2).

**Figure 3:**
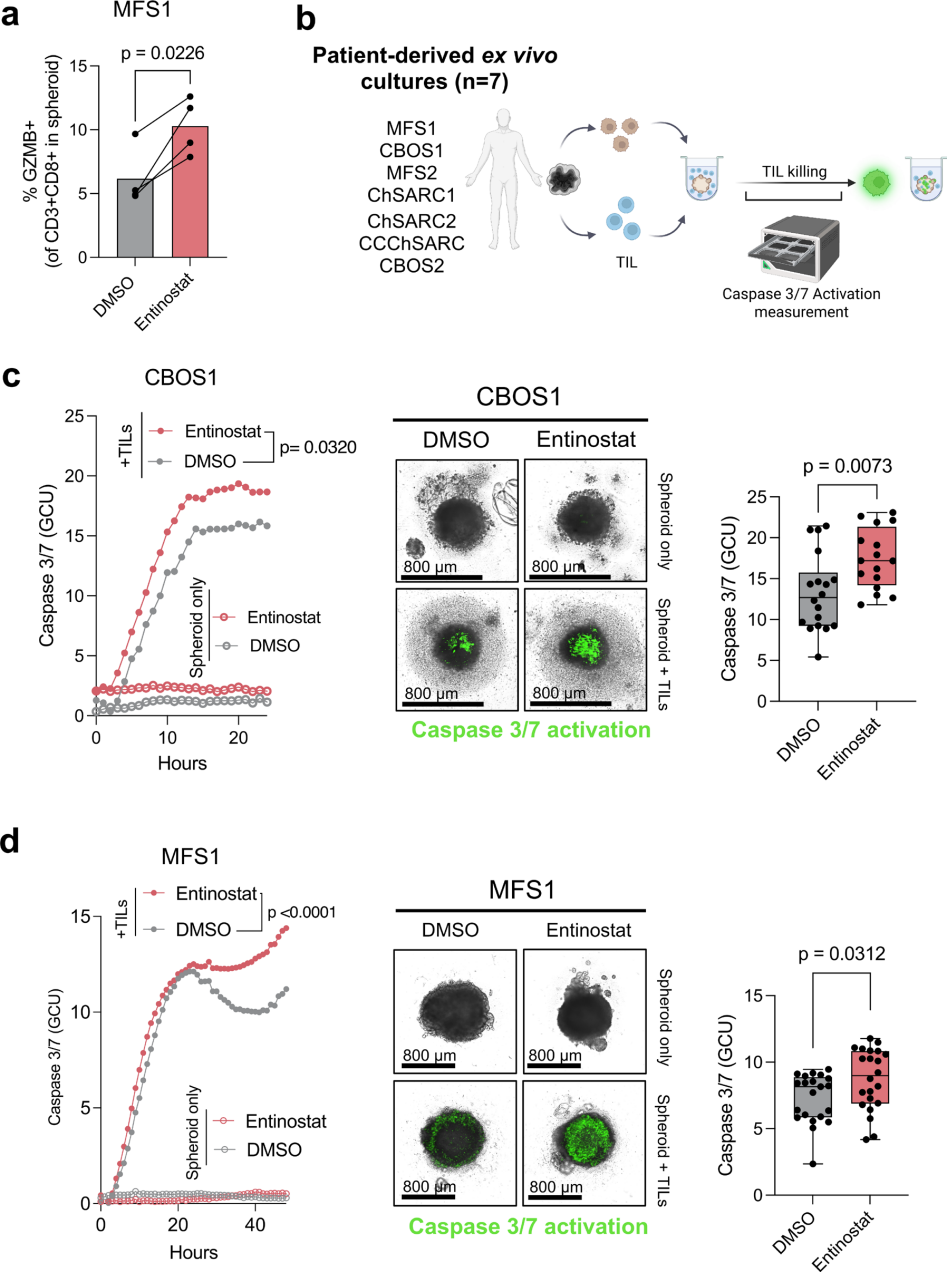
H3K27ac epigenetic modulation enhances TIL activation and effector function. (**a**) Frequency of granzyme B positive CD8^+^ T cells in MFS1-spheroids pretreated with or without 1 µM entinostat and after 48 hours of co-culture. (**b**) Experimental design of TIL killing activity in patient-derived tumor spheroids. (**c**) (Left) Caspase3/7 GCU within CBOS1 spheroid area, n=3 independent experiments, with five to six spheroids in each experiment and condition. (Middle) Representative image of Caspase 3/7 green fluorescence in CBOS1 spheroids at 12 hours of culture. (Right) Caspase 3/7 GCU at 12 hours of co-culture. Each dot represents a single CBOS1 tumor spheroid. (**d**) (Left) Caspase3/7 GCU within MFS1 spheroid area, n=3 independent experiments, with 4–6 spheroids in each experiment and condition. (Middle) Representative image of Caspase 3/7 green fluorescence in MFS1 spheroids at 12 hours of culture. (Right) Caspase 3/7 GCU at 12 hours of co-culture. Each dot represents a single MFS1 tumor spheroid. P values for (**a**), were calculated by paired t-test and two-way ANOVA or unpaired t-test for (**c and d**). ANOVA, analysis of variance; DMSO, dimethyl sulfoxide; GCU, green calibrated units; TIL, tumor-infiltating lymphocyte

While the infiltration of different T-cell subsets, including effector (T_EM_), central (T_CM_), and tissue-resident memory (T_RM_) T cells, impacts survival in many cancers, their role in sarcoma remains poorly studied.^15 16^ Analysis of the TCGA-SARC and TARGET-OS cohorts revealed that CD8^+^ T_RM_ cells, T_CM_, T_EM_, and effector T cells (T_EFF_), were associated with improved overall survival (figure 3h and online supplemental figure 3c,d). In ex vivo patient-derived cultures, entinostat-treated tumors displayed an enrichment in CD8^+^ T_RM_ cells. In contrast, no difference in the frequency of other memory cell populations was observed in spheroid-infiltrating TIL (figure 2i,j and online supplemental figure 3e,f). Collectively, these results show that entinostat-mediated permissive epigenetic state promotes TIL recruitment and T_RM_ enrichment in patient-derived sarcoma models.

### Entinostat-induced epigenetic state enhances TIL-mediated cytotoxicity in patient-derived sarcoma spheroids

We next examined whether increased infiltration of autologous TIL into H3K27ac-high tumors translated into enhanced cytotoxicity. In patient-derived ex vivo spheroid-TIL co-cultures, entinostat treatment resulted in significantly higher frequencies of granzyme B (GZMB)^+^CD8^+^ TIL (figure 3a). Given these results, the ability of entinostat to enhance TIL-mediated tumor killing was investigated by evaluating caspase-3/7 activation (figure 3b). Across multiple independent patient-derived ex vivo spheroid models, H3K27ac induction by entinostat led to increased TIL-mediated killing, as markedly observed by enhanced caspase-3/7 activation (figure 3c,d and online supplemental figure 4a–e). Together, these findings demonstrate that epigenetic H3K27ac remodeling by entinostat enhances the functional capacity of autologous TIL in patient-derived sarcoma spheroids, thereby increasing tumor cell killing.

### H3K27ac epigenetic modulation by entinostat increases infiltration of T cells in vivo

**Figure 4:**
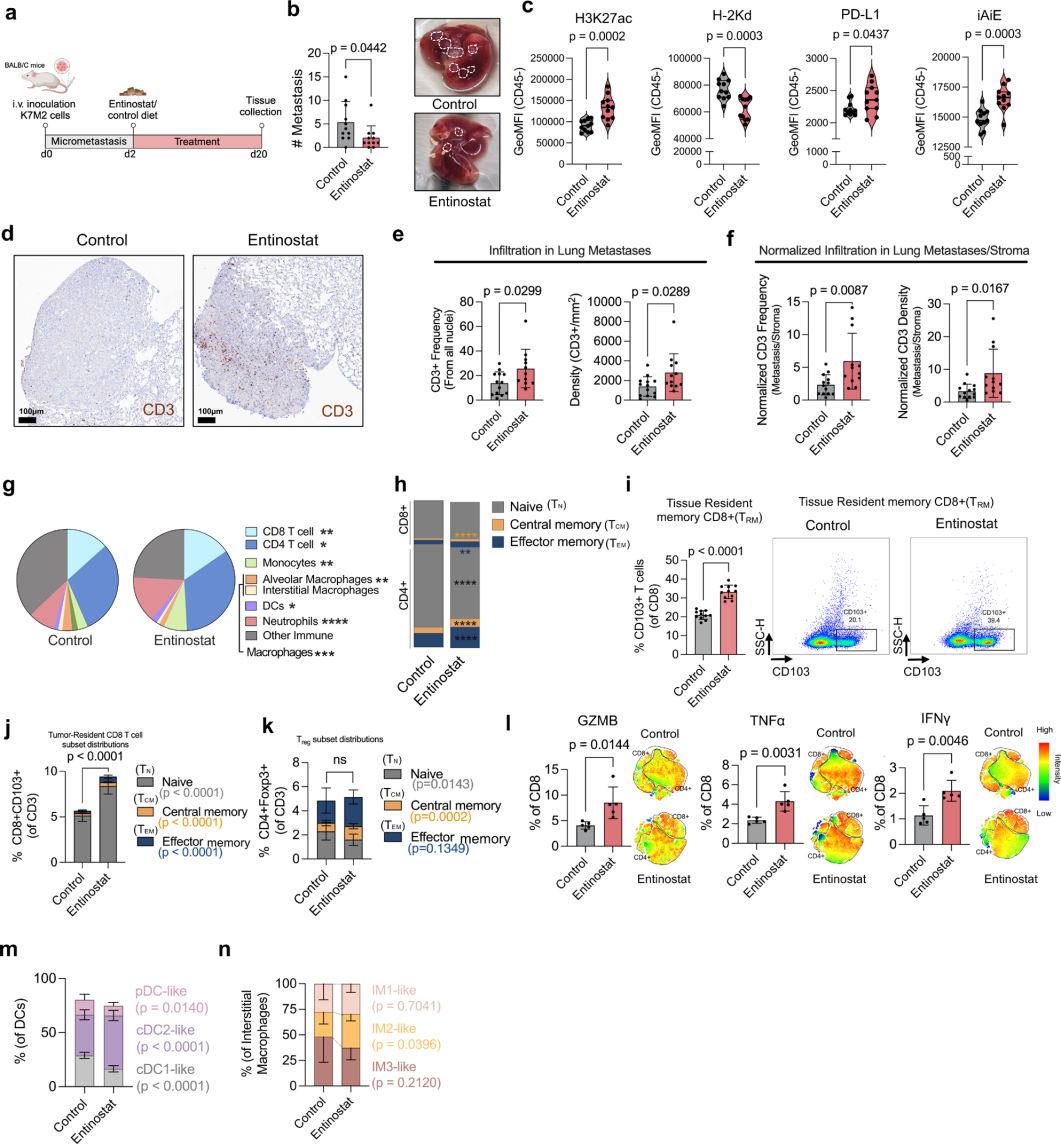
Entinostat induces T-cell activation and infiltration of memory and tissue resident T cells in vivo. (**a**) Design of in vivo experiments. (**b**) (Left) Number of metastases in the lungs of control and entinostat-treated mice. (Right) Representative images of lungs from control and entinostat mice with highlighted metastatic foci. (**c**) GeoMFI of H3K27ac, H-2Kd, PD-L1, and iAiE in lung metastasis gated on CD45^−^ cells. (**d**) Representative immunohistochemistry of CD3^+^ cells in metastatic lesions. (**e**) (Left) CD3^+^ cell frequency and (Right) density per mm^2^ in lung metastases, and (**f**) normalized to adjacent healthy lung stroma. Each dot represents one metastatic lesion. (**g**) Frequencies of immune populations in the lung. (**h**) Distribution of naïve (CD62L^+^CD44^−^), central (CD62L^+^CD44^+^), and effector memory (CD62L^−^CD44^+^), CD4^+^ and CD8^+^ T cells in the lung. (**i**) (Left) Frequency of T_RM_, defined as CD103^+^ of CD3^+^/CD8^+^ cells, and (Right) Representative flow cytometry plot of CD8^+^ T_RM_ from the lung. (**j**) Distribution of naïve, central, and effector memory CD8^+^ T_RM_ cells in the lung. (**k**) Frequency of CD4^+^/Foxp3^+^ T regulatory cells in the lung. (**l**) Frequencies of GZMB, TNFα, and IFNγ positive CD8^+^ T cells in the lung. Representative t-SNE heatmaps of CD4 and CD8^+^ T cells for each marker where higher intensity is depicted with red color. (**m**) Frequencies of cDC1-like, cDC2-like, and pDC-like from DCs in the lung. (**n**) Frequencies of IM, IM1-like, IM2-like, and IM3-like in the lung. Experiments were performed with (n=11) mice per group and each dot represents one individual mouse. Unpaired t-tests were used to test for significance with *p<0.05, **p<0.01, ***p<0.001, ****p<0.0001. cDC1, conventional type 1 DC; cDC2, conventional type 2 DC; DC, dendritic cell; GZMB, granzyme B; IFN, interferon; IM, interstitial macrophages; ns, non-significant; pDC, plasmacytoid DC; t-SNE, t-distributed stochastic neighbor embedding; TNF, tumor necrosis factor.

To examine if H3K27ac levels increased T-cell infiltration in vivo, the K7M2 pulmonary metastatic OS model was used (figure 4a). In vitro, K7M2 cells cultured as spheroids showed higher H3K27ac levels on exposure to entinostat (online supplemental figure 5a). In vivo, despite no difference in lung weight between entinostat-treated and control-treated mice, a significantly reduced number of metastases along with reduced spleen weight was observed in entinostat-treated mice (figure 4b and online supplemental figure 5b,c). In addition to higher levels of H3K27ac, tumor cells from entinostat-treated mice also showed higher levels of PD-L1, MHC class II (iAiE), but lower levels of MHC class I (H-2Kd) (figure 4c, online supplemental figure 5d, and online supplemental methods figure 3).

To assess whether entinostat-induced H3K27ac upregulation led to increased T-cell infiltration, immunohistochemistry (IHC) for CD3 was performed (online supplemental methods figure 4a,b). Both CD3^+^ frequency and density (cells/mm^2^) were significantly higher within metastatic pulmonary lesions of treated mice compared with control mice (figure 4d,e). When normalized to T-cell infiltration in the lung stroma, an even more substantial difference was observed (figure 4f). Moreover, within the lung tissues, both CD8^+^ and CD4^+^ T-cell frequencies were higher in entinostat-treated mice (figure 4g).

**Figure 5:**
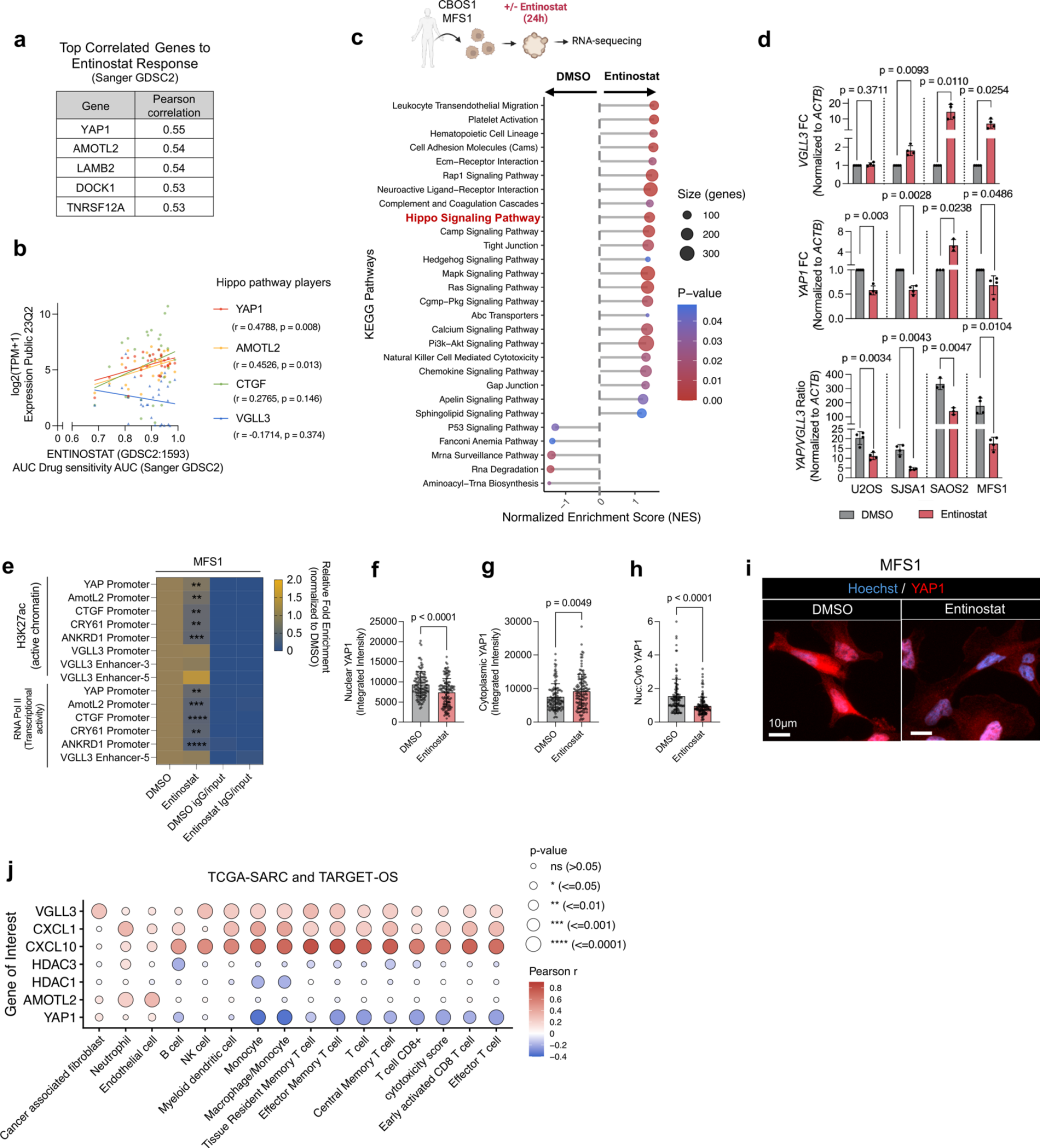
Entinostat modulates YAP1 and VGLL3 chromatin accessibility, expression, and protein localization. (**a**) Pearson correlation of the top five genes that correlate with response to entinostat in cell lines from the Cancer Cell Line Encyclopedia based on AUC read-out (Sanger (GDSC2)). (**b**) Entinostat response AUC Sanger (GDSC2) in sarcoma cell lines and its association with log2(TPM+1) gene expressions for Hippo pathway genes. (**c**) KEGG enriched pathways of CBOS1 and MFS1 spheroids. (**d**) FC gene expression by qRT-PCR of *VGLL3* (top) and *YAP1* (middle) between entinostat and DMSO treatment in different tumor spheroids (n=6 for each experimental condition). *YAP1/VGLL3* ratio (bottom), normalized to *ACTB*. (**e**) Relative fold enrichment normalized to DMSO by chromatin immunoprecipitation of H3K27ac depicting active chromatin and RNA Pol II as a measure of transcriptional activity in MFS1 spheroids (n=4). (**f–i**) Microscopic evaluation of YAP1 in MFS1 tumors on entinostat treatment showing (**f**) nucleus YAP1 mean intensity, (**g**) cytoplasmic YAP1 mean intensity, (**h**) nucleus to cytoplasm YAP1 ratio, and (**i**) representative confocal image. P values are calculated by Welch’s t-test. (**j**) Correlation plot of immune cells and genes of interest in the TCGA-SARC and TARGET-OS cohorts (n=342). Pearson r is represented by the color, and the corresponding p value by bubble size. ACTB, actin B; AMOTL2, angiomotin-like 2; AUC, area under the curve; CTGF, connective tissue growth factor; CXCL, C-X-C motif chemokine ligand; DMSO, dimethyl sulfoxide; FC, fold change; HDAC, histone deacetylases; KEGG, Kyoto Encyclopedia of Genes and Genomes; NK, natural killer; OS, osteosarcoma; qRT-PCR, quantitative reverse transcription polymerase chain reaction; RNA Pol II, RNA polymerase-II binding; TARGET, Therapeutically Applicable Research to Generate Effective Treatments; TCGA-SARC, The Cancer Genome Atlas – Sarcoma; TPM, Transcripts per million; VGLL3, vestigial-like family member 3; YAP1, yes-associated protein 1.

On analysis of T-cell subsets, epigenetic modulation of H3K27ac significantly increased T_CM_ and T_EM_ subsets in CD8^+^ and CD4^+^ populations within the lung (figure 4h). As observed in human ex vivo spheroids, entinostat treatment significantly elevated the frequency of pulmonary CD8^+^ T_RM_ cells (figure 4i and online supplemental methods figure 5a). Moreover, analysis of T_RM_ populations revealed an increase in the frequency of T_EM_, T_CM_, and naive T cells within the CD8^+^ T_RM_ compartment (figure 4j). The frequency of regulatory T cells (T_reg_) remained unchanged in lungs of treated mice, however, there was an increase of T_CM_ within this population (figure 4k). With regards to T-cell function, entinostat-treated mice had significantly higher frequencies of GZMB, tumor necrosis factor-α (TNFα), and interferon-γ (IFNγ) CD8^+^ T cells, but no activation of CD4^+^ T cells was observed within the lung TME (figure 4l and online supplemental figure 5e,f, and online supplemental methods figure 5b).

On analysis of peripheral sites, the frequency of total CD4^+^ and CD8^+^ T cells in the spleen was higher in entinostat-treated mice, specifically of CD8^+^ T_CM_, and CD4^+^ T_EM_ (online supplemental figure 5g,h and online supplemental methods figure 5c). Moreover, a significant reduction of T_reg_ cells was found in the spleen on treatment with entinostat (online supplemental figure 5i,j). In the bone marrow, no difference in total CD8^+^ T cells was observed in entinostat-treated mice. However, CD8^+^ naïve subset was significantly increased, while the proportion of CD8^+^ T_EM_ and naïve CD4^+^ T cells were reduced (online supplemental figure 5k,l).

Together, these results show that entinostat-mediated inhibition of HDAC1/3 in OS induces significant changes in T-cell compartments within the lung TME and at peripheral sites in vivo. Specifically, entinostat increases H3K27ac levels, thereby promoting T-cell infiltration, activation, memory, and resident T-cell populations.

### Entinostat reprograms the myeloid compartment toward a pro-inflammatory state in vivo

**Figure 6:**
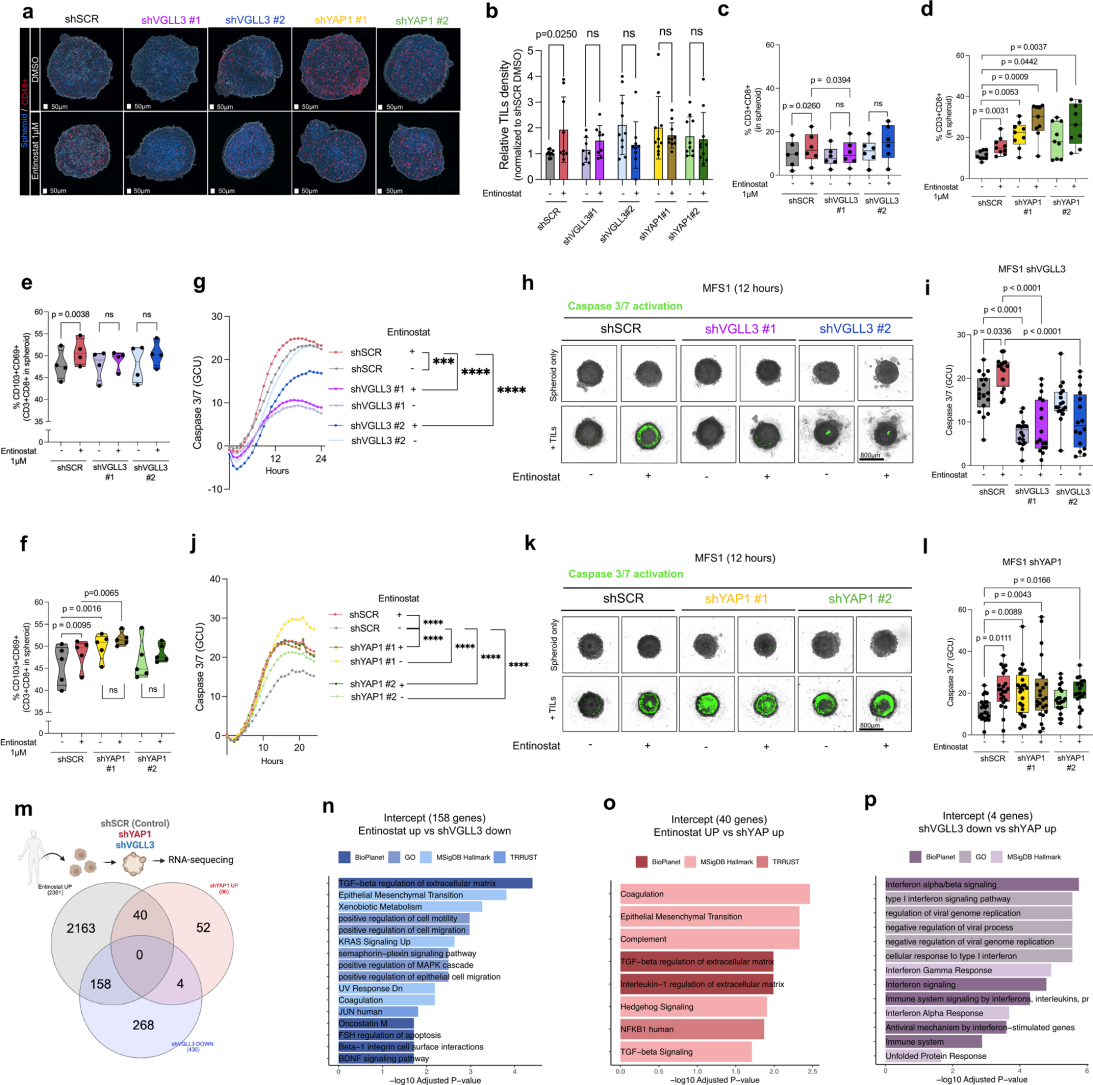
VGLL3 and YAP1 oppositely regulate entinostat-mediated TIL infiltration. (**a**) Representative images of TIL infiltration into MFS1 spheroids. (**b**) Relative MFS1-TIL density normalized to shSCR DMSO control. Each dot represents one spheroid. P values were calculated by two-way ANOVA statistical test. (**c–d**) Flow cytometry analysis of CD8^+^ T-cell infiltration in shVGLL3 or shYAP1. (**e–f**) Flow cytometry analysis of CD8^+^ T_RM_ TIL infiltration into MFS1 tumor spheroids. (**g–l**) Caspase 3/7 GCU in untreated and entinostat-treated VGLL3 or YAP1 knockdown MFS1 spheroids in the presence of autologous TIL. Values are normalized to respective MFS1 spheroids alone. Mean GCU values of n=3 or n=4 of independent experiments with five to six spheroids per condition are shown for shVGLL3 or shYAP1, respectively. (**h and k**) Representative fluorescence images at 12 hours. (**i and j**) Quantification of Caspase 3/7 in MFS1 spheroids at 12 hours in the presence of autologous TIL. Each dot represents one spheroid/TIL culture. (**m**) Sequencing conducted in shSCR, shVGLL3, and shYAP1 MFS1 spheroids (n=3 samples per condition). Identification of shared transcriptional modulation by intersecting differentially upregulated genes in entinostat, shYAP1, while differentially downregulated in shVGLL3. (**n**) Pathway enrichment analysis of intercepted DEG (158) upregulated by entinostat and downregulated by shVGLL3. (**o**) Pathway enrichment analysis of intercepted DEG (40) upregulated by entinostat and shYAP1. (**p**) Pathway enrichment analysis of intercepted DEG (4) upregulated by shYAP1 but downregulated in shVGLL3. ANOVA, analysis of variance; DEG, differentially expressed gene; DMSO, dimethyl sulfoxide,;GCU, green calibrated units; TIL, tumor-infilgrating lymphocyte; T_RM_, tissue-resident memory T cell; VGLL3, vestigial-like family member 3; YAP1, yes-associated protein 1.

Given the importance of myeloid cells in regulating antitumor immune responses, tumor-bearing mice were examined for changes in myeloid cell compartments following entinostat treatment. Myeloid cell states were evaluated by analyzing the expression of major histocompatibility complex (MHC) class II and CD86 which reflect antigen presentation and costimulatory capacity respectively, and arginase-1 (Arg1) to evaluate immunosuppressive polarization across myeloid subsets. Within the lung TME, several alterations were observed; these manifested as increased frequencies of neutrophils, monocytes, and dendritic cells (DCs), while decreased proportions of macrophages, specifically alveolar macrophages (AM) (figure 4g and online supplemental methods figure 6). Analysis of the DC compartment revealed an increase in conventional dendritic cell (cDC)2-like cells, accompanied by reductions in cDC1-like and plasmacytoid (pDC)-like cells (figure 4m). Among lung interstitial macrophages (IM), entinostat increased the frequency of IM2-like macrophages, a population associated with heightened pro-inflammatory activity relative to IM1-like macrophages (figure 4n). Beyond compositional changes, entinostat increased the expression of CD86 on cDC2-like, macrophages, IM1-like, and monocytes. Furthermore, MHC class II was upregulated in monocytes and pDCs-like cells, as well as Arg1 in AM-like cells (online supplemental figure 6a,b).

Systemic immune compartments mirrored these trends. Within the spleen, entinostat augmented the frequencies of total DC, cDC2-like, pDC-like cells, while decreasing cDC1-like cells (online supplemental figures 5g and 6c). Within the bone marrow, the frequencies of monocytes, neutrophils, and DC were increased by the treatment, while the frequency of cDC1-like cells was decreased (online supplemental figures 5k and 6d). Furthermore, higher CD86-positive macrophages and monocytes were observed in the spleen and bone marrow of entinostat-treated mice. In contrast, reduced frequencies of Arg1-positive macrophages and monocytes were observed in the spleens of treated mice (online supplemental figure 6e,f).

To assess whether these effects were conserved in human cells, human CD14^+^ isolated cells were exposed to entinostat. Consistent with the murine data, entinostat upregulated CD86 expression and reduced indoleamine 2,3-dioxygenase (IDO) levels. However, no significant effects were observed on Arg1 or MHC class II (online supplemental figure 6g). Taken together, these results demonstrate that modulation of H3K27ac by entinostat alters the frequencies of myeloid cell populations in OS, suggesting an overall heightened inflammatory state within the lung TME and peripheral sites.

### Entinostat-induced epigenetic remodeling modulates the Hippo pathway by upregulating VGLL3 and downregulating YAP1

To investigate the underlying mechanisms of enhanced T-cell infiltration in H3K27ac high tumors, the response to entinostat treatment was evaluated across multiple cell lines from the Cancer Cell Line Encyclopedia.^17^ This analysis revealed that the Hippo pathway-related molecules *YAP1* and angiomotin-like 2 (*AMOTL2*) were the top genes correlating with resistance to entinostat (figure 5a and online supplemental figure 7a,b). Consistently, both *YAP1*, and *AMOTL2*, correlated with resistance to entinostat treatment specifically in STS and OS cell lines (figure 5b). Likewise, a similar pattern was observed for connective tissue growth factor (*CTGF*), a well-characterized downstream target of YAP1 (figure 5b and online supplemental figure 7c). In the inactive state of the Hippo pathway, YAP1 binds to TEAD transcription factors, promoting gene expression and influencing several key biological processes including cell proliferation, migration, invasion, and epithelial to mesenchymal transition. Additionally, VGLL3 competes with YAP1 for TEAD binding, modulating TEAD-driven transcription and opposing YAP1’s effects. While the expression of *VGLL3* showed a positive correlation with entinostat resistance across all cancers, it negatively correlated with resistance to entinostat treatment specifically in sarcoma cell lines, suggesting a context-dependent role in entinostat response and the involvement of the Hippo pathway in sarcoma (figure 5b and online supplemental figure 7d).

To confirm these observations, RNA-seq of ex vivo patient-derived tumor spheroids was performed (online supplemental figure 7e). Indeed, Gene-Set Enrichment Score (GSEA) of Kyoto Encyclopedia of Genes and Genomes (KEGG) pathways revealed alterations in the Hippo pathway on entinostat treatment. Furthermore, upregulation of immune-related pathways, including cell adhesion processes, and leukocyte transendothelial migration was observed in entinostat-treated compared with untreated tumor spheroids (figure 5c, online supplemental figure 7f). Differential gene expression analysis revealed higher expression of genes indicative of an active Hippo pathway, with *VGLL3* being among the most significantly upregulated (online supplemental figure 7g,h). As corroborated by quantitative reverse transcription polymerase chain reaction (qRT-PCR), exposure to entinostat resulted in higher *VGLL3*, while reducing *YAP1* and the *YAP1:VGLL3* ratio (figure 5d and online supplemental figure 7i–k). Together, these data show that entinostat treatment results in a downregulation of *YAP1* and a shift towards *VGLL3*.

### Entinostat remodels chromatin accessibility and YAP1 localization

To further interrogate the involvement of the Hippo pathway on treatment with entinostat, H3K27ac was investigated as an active chromatin marker at promoter and enhancer regions, together with RNA polymerase II (RNA Pol II) binding to measure transcriptional activation. Indeed, a significant decrease in the abundance of H3K27ac and RNA Pol II binding on entinostat treatment in the promoter of *YAP1*, *AMOTL2,* and downstream YAP-driver genes (*CTGF, CRY61, ANKRD1*) was observed. On the other hand, although not significant, induction of H3K27ac and RNA Pol II binding was observed in *VGLL3* enhancers and promoters (figure 5e). Moreover, confocal microscopy of YAP1 protein levels revealed a significantly reduced nuclear-to-cytoplasmic ratio in entinostat-treated tumors, indicating minor nuclear presence and decreased YAP1 function (figure 5f–i).

On analysis of cancers with *VGLL3* amplification within the TCGA data cohort, only amplified sarcoma cases showed higher CD8^+^ T-cell infiltration compared with cases with normal *VGLL3* levels (online supplemental figure 7l,m). Additionally, data from TCGA-SARC and TARGET-OS demonstrated a positive correlation between *VGLL3* expression and various T-cell memory populations, including T_RM_, T_EM_ and T_CM_, clustering together with the inflammatory chemokines *CXCL1* and *CXCL10*. In contrast, *YAP1*, *HDAC1*, *HDAC3* and *AMOTL2*, negatively correlated with CD8^+^ T-cell infiltration (figure 5j). To investigate the clinical relevance of YAP1 and VGLL3, patients with OS and STS were categorized into high and low *YAP1* and *VGLL3* gene expression levels. Patients with *YAP1*^low^/*VGLL3^h^*^igh^ tumors showed significantly prolonged survival compared with patients with *YAP1*^high^/*VGLL3*^low^ tumors (online supplemental figure 7n). Together, these results show that entinostat modulates the Hippo pathway by downregulating YAP1 and upregulating VGLL3, and that these alterations are associated with enhanced immune activation and a shift toward a more inflammatory TME.

### VGLL3 and YAP1 oppositely regulate entinostat-mediated TIL infiltration and function

**Figure 7:**
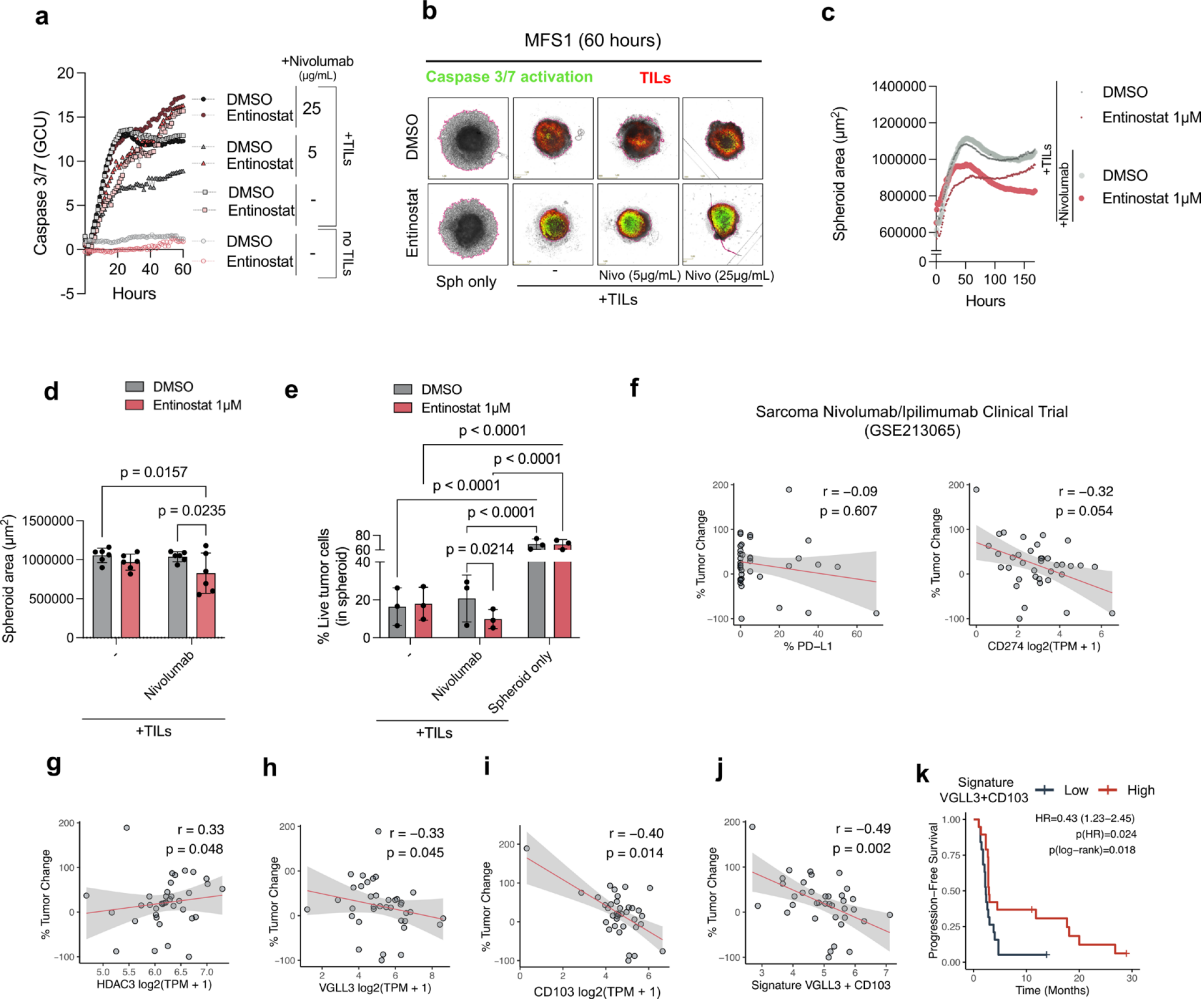
Entinostat primes sarcoma spheroids for PD-1 blockade, and VGLL3/CD103 predict clinical benefit to ICI in Sarcoma. (**a**) TIL-mediated killing of MFS1 spheroids in the presence or absence of entinostat (1 µM) and nivolumab at5 or 25 µg/mL over time in MFS1 spheroids. (**b**) Representative spheroid images at 60 hours of culture. (**c**) Spheroid area throughout a 7-day co-culture with TIL. (**d**) Spheroid area measured at the end of the co-culture (day 7). (**e**) Frequency of live tumor cells within spheroids after 7 days of culture. (**f**) Spearman correlation of percentage change from baseline in the sum of target lesion diameters against pre-ICI %PD-L1 protein levels by IHC (left), or PD-L1 gene levels (CD274) log2(TPM+1) in 37 patients with sarcoma treated with nivolumab/ipilimumab (GSE213065). (**g–i**) Spearman correlation of gene expression log2(TPM+1) against % change in tumor size in patients with nivolumab/ipilimumab sarcoma. (**j**) Correlation of tumor change and VGLL3/CD103 signature, calculated as the mean of the log2(TPM+1) of each gene, VGLL3 and ITGAE(CD103). (**k**) Kaplan-Meier curves for progression-free survival of 38 patients with sarcoma stratified by median expression of VGLL3 and CD103 signature (High (n=19 patients) vs Low (n=19 patients)). P(log-rank) is the log-rank test p value; HR (95% CI) and p(HR) are from a univariate Cox model comparing High versus Low. DMSO, dimethyl sulfoxide; GCU, green calibrated units; HDAC, histone deacetylases; ICI, immune checkpoint inhibitor; IHC, immunohistochemistry; PD-1, programmed cell death protein-1; PD-L1, programmed death-ligand 1; TIL, tumor-infiltrating lymphocyte; TPM, transcripts per million, VGLL3, vestigial-like family member 3.

To test whether VGLL3 and YAP1 impact on entinostat-mediated T-cell infiltration, TILs were co-cultured with YAP1 or VGLL3 short-hairpin knockdown (sh) patient-derived sarcoma spheroids (online supplemental methods figure 7a–d). ShVGLL3 abrogated entinostat-induced increase in total and CD8+T_RM_ TIL infiltration. In contrast, YAP1 knockdown resulted in an elevated baseline infiltration of total T cells and T_RM_ cells, and treatment with entinostat did not increase T-cell infiltration in shYAP1 tumors (figure 6a-f; online supplemental figure 8a,b). Functionally, VGLL3 knockdown abolished entinostat-induced increase in TIL-mediated killing, whereas YAP1 knockdown increased baseline killing (figure 6g–l; online supplemental figure 8c–e). To substantiate these results, the US Food and Drug Administration (FDA)-approved drug verteporfin (VP), which acts as a YAP1-TEAD binding inhibitor, was used. Indeed, treatment with VP increased overall CD8^+^ TIL and T_RM_ infiltration (online supplemental figure 8f–h). Moreover, while treatment with VP alone did not impact tumor cell viability, VP sensitized tumor cells to TIL-mediated killing (online supplemental figure 8i). Overall, our results demonstrate that VGLL3 is required for entinostat-mediated enhancement of TIL infiltration and function, while YAP1 acts as a suppressor whose inhibition mimics entinostat immune effects.

### YAP1 and VGLL3 exhibit divergent transcriptional programs in entinostat-driven epigenetic reprogramming of the tumor microenvironment

To identify molecular cues differentially regulated by YAP1 and VGLL3, RNA-seq was performed on shSCR, shVGLL3, and shYAP1 tumor spheroids (online supplemental figure 9a). Differentially expressed genes (DEGs) were compared with entinostat-induced DEGs to define shared transcriptional programs. This analysis identified 158 genes upregulated by entinostat but downregulated in shVGLL3 spheroids, reflecting VGLL3-associated repression, and 40 genes upregulated by both entinostat and in shYAP1 spheroids, suggesting YAP1-dependent induction. Only four genes showed opposing regulation between shVGLL3 and shYAP1 (figure 6m). Gene Set Enrichment Analysis of these gene intercepts showed enrichment of transforming growth factor-beta (TGF-β) pathways related to extracellular matrix organization (figure 6n,o) and immune-related pathways, like NF-kB, interleukin-mediated responses, and type I interferon signaling (figure 6o,p). Conversely, analysis of downregulated intercepts by entinostat and shYAP1, but upregulated by shVGLL3 (online supplemental figure 9b), were implicated in the suppression of insulin-like growth factor signaling and broad transcriptional networks (online supplemental figure 9c–f). Overall, these findings confirm that YAP1 and VGLL3 exert opposing regulatory effects on transcriptional programs that control immune signaling and ECM organization, and that entinostat treatment partially recapitulates VGLL3-associated transcriptional signatures while antagonizing YAP1-driven pathways.

### VGLL3/T_RM_ signatures predict responses to anti-PD-1 therapy in sarcoma

To extend the findings of upregulated tumor programmed death-ligand 1 (PD-L1) expression on treatment with entinostat, targeting programmed cell death protein-1 (PD-1) (nivolumab) was explored in patient-derived sarcoma spheroids. Real-time analysis revealed no effect of nivolumab alone on TIL-mediated killing. However, a higher caspase-3/7 activity on tumor cells was observed when combining nivolumab with H3K27ac enhancement by entinostat (figure 7a, b). Prolonged co-culture showed reduced spheroid size and decreased frequency of live cells when combining entinostat with nivolumab (figure 7c–e). To assess whether entinostat-regulated genes were involved in clinical response, a publicly available database of 38 patients with STS receiving ICI treatment (GSE213065)^18^ was analyzed. Tumor progression did not correlate with PD-L1, *CD274, CXCL10, HDAC1, YAP1, and AMOTL2* expression (figure 7f and online supplemental figure 10). In contrast, *HDAC3, VGLL3,* and *ITGAE* correlated with tumor regression (figure 7g–i). Furthermore, patients with high *VGLL3* and *ITGAE* expression showed increased tumor regression and prolonged overall survival, highlighting the potential of this signature to predict responses to ICI in sarcoma (figure 7j,k). Together, these results support the combination of entinostat treatment with anti-PD-1 immunotherapy, and that VGLL3/T_RM_ signatures predict response to anti-PD-1-treatment outcomes in STS.

## Discussion

Infiltration of T cells is associated with improved prognosis and response to ICI across several solid cancers.^19 20^ Sarcomas, however, are characterized by extensive epigenetic dysregulation and generally display limited immune infiltration. In this study, we investigated whether epigenetic modulation directly influences the inflammatory landscape of sarcoma. Previous work examining the immunomodulatory effects of epigenetic therapies has mainly relied on murine models and commercial cell lines.^21^,^25^ Also, sarcoma preclinical research has primarily focused on pan-HDAC inhibitors, which exhibit significant cardiotoxicity.^26^ Here, using patient-derived ex vivo spheroids and in vivo metastatic OS models, we demonstrate that H3K27ac levels play a crucial role in shaping the immune landscape in sarcoma. Specifically, selective inhibition of HDAC1/3 activity by entinostat, a compound with favorable safety profiles in sarcoma,^27 28^ increased H3K27ac levels and consistently enhanced CD8^+^ T-cell infiltration across experimental systems.

While immune infiltration is key to antitumor immunity, sustained control relies on the functional activity of these cells. Various memory T-cell subsets, including T_RM_, display cytotoxic function and are associated with improved clinical outcomes in several cancers.^15^ In sarcomas, however, their role remains unexplored.^16 29 30^ T_RM_ are characterized by the expression of CD103, sometimes combined with CD69 or CD49a, with marker usage being context dependent.^31^ CD103 forms an integrin heterodimer with β7 that binds to E-cadherin, leading to cell adhesion, migration, and retention in specific tissues.^29^ Our results across clinical datasets demonstrate that T_RM_ can serve as a biomarker of sarcoma prognosis and immunotherapy response, and we show that H3K27ac modulation by entinostat enriches for CD103+T_RM_ infiltration both in vivo and in ex vivo patient-derived models. Accordingly, we interpret enrichment of CD103^+^ cells as indicative of a resident-memory–like compartment rather than definitive tissue residency or sole mediators of tumor control. Moreover, entinostat also increased the presence of T-cell memory populations in vivo. In parallel, we describe that H3K27ac modulation reprogrammed the myeloid compartment to a pro-inflammatory state, increasing IM2-like and DC infiltration. Moreover, we observed upregulation of costimulatory molecules, such as CD86, resulting from direct epigenetic reprogramming within the myeloid compartment.

Mechanistically, we identify modulation of the Hippo pathway as a central determinant of the immunostimulatory effects induced by entinostat. Entinostat treatment resulted in downregulation of YAP1 and concomitant upregulation of VGLL3, two transcriptional regulators that compete for TEAD binding and exert opposing effects on gene expression programs.^32^ Silencing YAP1 recapitulated the effects of entinostat in enhancing CD8^+^ T cell and T_RM_ infiltration, thereby promoting tumor cell killing, whereas VGLL3 knockdown reversed these entinostat-mediated effects. Consistent with this, we demonstrate that YAP1 targeting with non-photoactivated VP induces effects similar to those of entinostat by enhancing CD8^+^ T cell and T_RM_ infiltration, as well as TIL function, identifying an additional immunomodulatory target for sarcomas.

Although VGLL3 amplification has been reported in sarcoma and linked to tumor-intrinsic transcriptional programs, its potential role in regulating antitumor immune responses has not been previously defined. Here, we demonstrate that VGLL3 expression correlates with CD8^+^ T cell and T_RM_ infiltration, and favorable ICI outcomes, establishing VGLL3 as a determinant of an immune-permissive TME. This aligns with earlier studies demonstrating that VGLL3 upregulates pro-inflammatory genes like IL-1α and is implicated in autoimmune diseases.^33^,^36^ Conversely, YAP1, which is highly activated in sarcomas,^37 38^ has been associated with tumor promotion,^39^ and its expression in ovarian cancer-associated fibroblasts has recently been found to suppress CD8^+^ T cell responses and to be higher in non-responders to chemotherapy.^40^

Consistent with prior clinical observations, entinostat as monotherapy has, so far, yielded only modest responses.^27 28^ Moreover, sarcomas show poor responses to ICI, and the lack of biomarkers complicates patient selection.^828 41^,^46^ This was also the case with our ex vivo model, where ICI alone had no effect on TIL infiltration or TIL-mediated tumor cell killing. Indeed, we found that combining entinostat with anti-PD1 sustained T cell-mediated cytotoxicity against patient-derived tumor spheroids over time. In line with these findings, recent studies in metastatic uveal melanoma,^47^ pancreatic cancer,^48^ breast cancer,^49^ and a limited number of sarcomas^50^ described improved responses in subsets of patients. For instance, it has been reported that patients with sarcoma who respond to ICI exhibit higher CD8^+^ T_EM_.^51^ Here, in a cohort of patients with sarcoma receiving ICI, we identified that high VGLL3/ITGAE (CD103) signatures before therapy predict clinical response, further supporting our findings, identifying potential biomarkers, and suggesting epigenetic pretreatment to elevate VGLL3 and T_RM_ infiltration for improved ICI responses.

In summary, we characterize that H3K27ac epigenetic modulation via entinostat impacts the Hippo pathway players VGLL3 and YAP1 to reprogram the TME, resulting in increased CD8^+^ T_RM_ cell infiltration and activation. Since GSEA showed upregulation of TGF-β regulation of extracellular matrix, and TGF-β is a key molecule for the development of T_RM_ cells,^52^ it provides a potential link to T_RM_ infiltration into Entinostat-treated tumors. Beyond T cells, our results demonstrate that entinostat induces significant changes in the myeloid compartment, consistent with an earlier study showing its ability to reprogram myeloid-derived suppressor cells (MDSCs) in breast and pancreatic cancers.^53^ Previous studies have shown that HDAC1/3 inhibition can upregulate the expression of tumor neoantigens.^54^ Along these lines, we observed alterations in antigen presentation pathways in tumors of Entinostat-treated mice, including increased MHC-II expression in myeloid populations and reduced MHC-I expression in tumor cells. Together, these findings suggest a complex remodeling of antigen presentation pathways in epigenetically permissive tumors and warrant further investigation into how entinostat influences antigen repertoire and presentation. While entinostat-mediated HDAC1/3 inhibition robustly increases H3K27 acetylation and is accompanied by immune remodeling, its effects on non-histone protein acetylation at higher doses should also be considered. Although most functional experiments in this study were conducted in OS models, complementary transcriptomic analyses and ex vivo patient-derived samples across multiple STS histologies support the broader relevance of these epigenetic-immune findings in different sarcoma subtypes. However, since sarcomas comprise a biologically heterogeneous group of malignancies, with OS differing substantially from STS in genomic architecture and immune context, larger and subtype-resolved studies would be needed. Collectively, our findings provide a strong rationale for incorporating epigenetic preconditioning with immunotherapy to improve outcomes in patients with sarcoma, warranting further investigation in combination therapies and the biomarker applicability of T_RM_ and H3K27ac to guide combination immunotherapy approaches.

## Supplementary material

10.1136/jitc-2025-014601 online supplemental file 1

10.1136/jitc-2025-014601 online supplemental file 2

## Data Availability

Data are available in a public, open access repository. Data are available upon reasonable request.
