# Supplementary material for: Epigenetic remodeling in sarcoma promotes T-cell infiltration via modulation of the Hippo pathway
Source: J Immunother Cancer. 2026 Apr 1;14(4):e014601. doi: 10.1136/jitc-2025-014601 (PMC13052610; doi:10.1136/jitc-2025-014601)
Supplement: online supplemental file 1 [file jitc-14-4-s001.pdf]

Supplementary Materials for:

Epigenetic remodeling in sarcoma promotes T cell infiltration via modulation of the Hippo pathway

Mireia Cruz De los Santos, Yi Chen, Amaia González de Zárate, Agnes Sorteberg, Honglei Zhao, Guillermo Vázquez-Cabrera, Neda Bigdeli, Solrun Kolbeinsdottir, Aarren Mannion, Lucas Baldran-Grooves, Shi Yong Neo, Stina L. Wickström, Jeroen Melief, Lars Holmgren, Nikolas Herold, Felix Haglund de Flon, Andreas Lundqvist.

<u>**Table of contents**</u>

Supplementary Figures:

- Supplementary Figure 1
- Supplementary Figure 2
- Supplementary Figure 3
- Supplementary Figure 4
- Supplementary Figure 5
- Supplementary Figure 6
- Supplementary Figure 7
- Supplementary Figure 8
- Supplementary Figure 9
- Supplementary Figure 10

Supplementary Methods Figures:

- Supplementary Methods Figure 1
- Supplementary Methods Figure 2
- Supplementary Methods Figure 3
- Supplementary Methods Figure 4
- Supplementary Methods Figure 5
- Supplementary Methods Figure 6
- Supplementary Methods Figure 7

Supplementary Tables:

- Supplementary Table 1
- Supplementary Table 2

Supplementary Methods

- Sarcoma tissue processing and tumor cell culture
- T cell expansion
- Lentiviral-induced knock-down of patient-derived ex vivo sarcoma cultures
- Real-time killing assay analysis
- Murine osteosarcoma model and tissue processing
- Immunohistochemistry staining and analysis of murine lung tissue
- Flow cytometry analysis
- Assessment of infiltration using 3D confocal imaging
- Chromatin Immunoprecipitation
- Western Blot
- RNA isolation
- Quantitative Real-Time PCR and analysis
- Confocal microscopy and analysis of YAP1
- Public patient cohort epigenome score analysis
- Histone-mark annotation and tumor-specific epi-PCG gene sets
- Signature scoring, clustering, and survival of epigenetic scores
- Pathway association and immune infiltration analyses of epigenetic scores
- Histone-deacetylase analysis of TARGET-OS and TCGA-SARC
- Entinostat sensitivity and cell line RNA expression
- Genomic amplifications in cancer and immune correlations
- Immune deconvolution and correlation with Hippo pathway genes
- Patient-derived spheroids RNA sequencing
- Immunotherapy response analysis
- Single-cell data analysis
- Statistics

Supplementary Materials Table:

- Supplementary Materials Table 1
- Supplementary Materials Table 2
- Supplementary Materials Table 3

Supplementary Figures

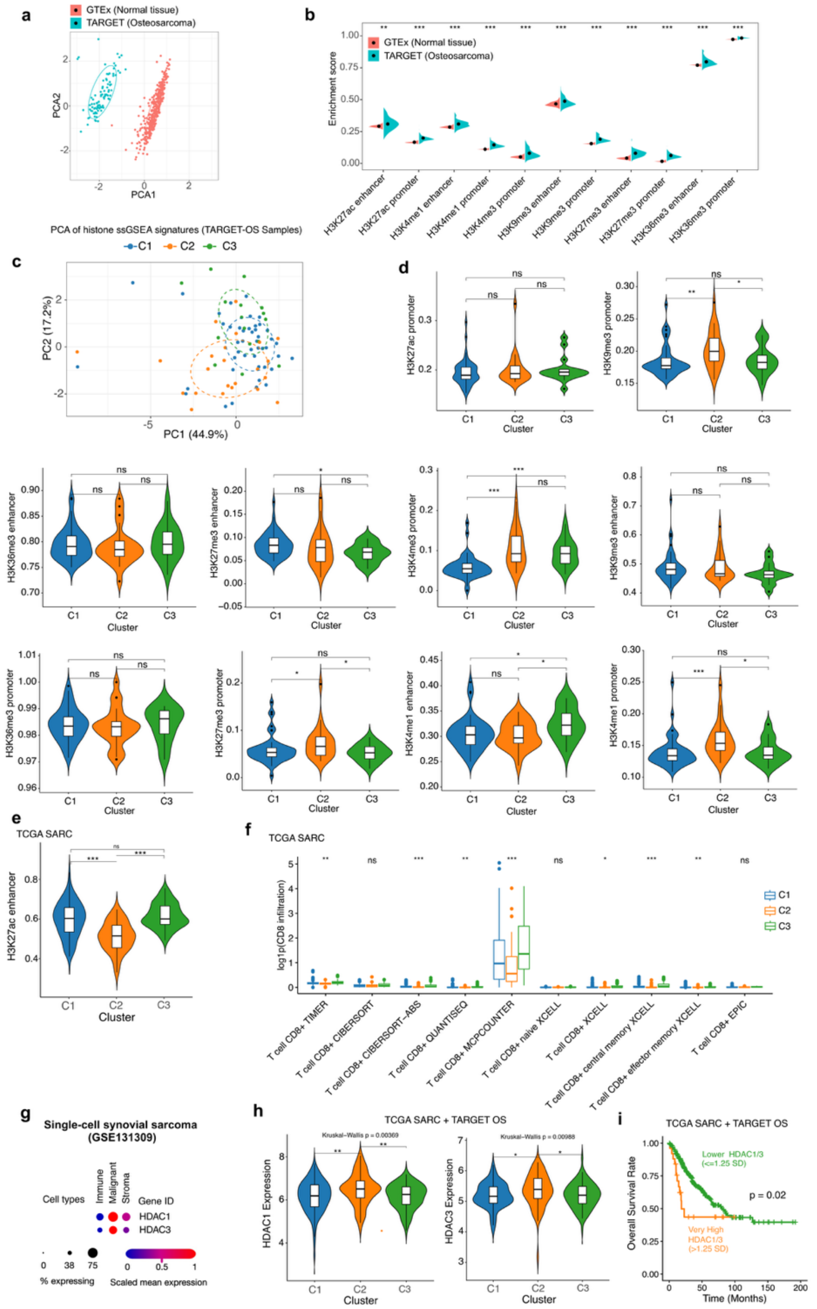

Supplementary Figure 1. Sarcoma transcriptomic analysis reveals an altered epigenome associated with a worse prognosis. a) Principal component analysis (PCA) of epigenome score of RNA sequencing data from 84 OS patients in the TARGET cohort (blue) compared to corresponding healthy tissue from GTEx (red). b) Enrichment of specific histone modifications in healthy tissue samples from GTEx and OS TARGET samples. c) TARGET-OS cohort PCA of histone ssGSEA signatures. d) Histone modification levels at enhancer and promoter regions across the three clusters (C1-C3) in TARGET-OS. e) H3K27ac ssGSEA signatures in TCGA-SARC and f) CD8 T cell infiltration deconvolution in TCGA-SARC clusters. C1 (n=120), C2 (n=52), C3 (n=86) g) scRNA-seq data from synovial sarcoma samples (GSE131309) (n=12). f-g) Kaplan Meier curves of combined TCGA SARC and TARGET OS. h) HDAC 1 and HDAC3 expression in clusters C1-C3 of TCGA-SARC and TARGET-OS patients. C1 (n=165), C2 (n=75), C3 (n=102). In f), patients were grouped into “Very High” (n=25) or ”Lower” (n=317) expression based on combined HDAC1-HDAC3 expression levels and using a cutoff of 1.25 SD (Standard deviation). Differences between curves are assessed using log-rank test. P-values = p<0.05 = *, p<0.01 = **, p<0.001 = ***, p<0.0001 = **** , ns (not significant).

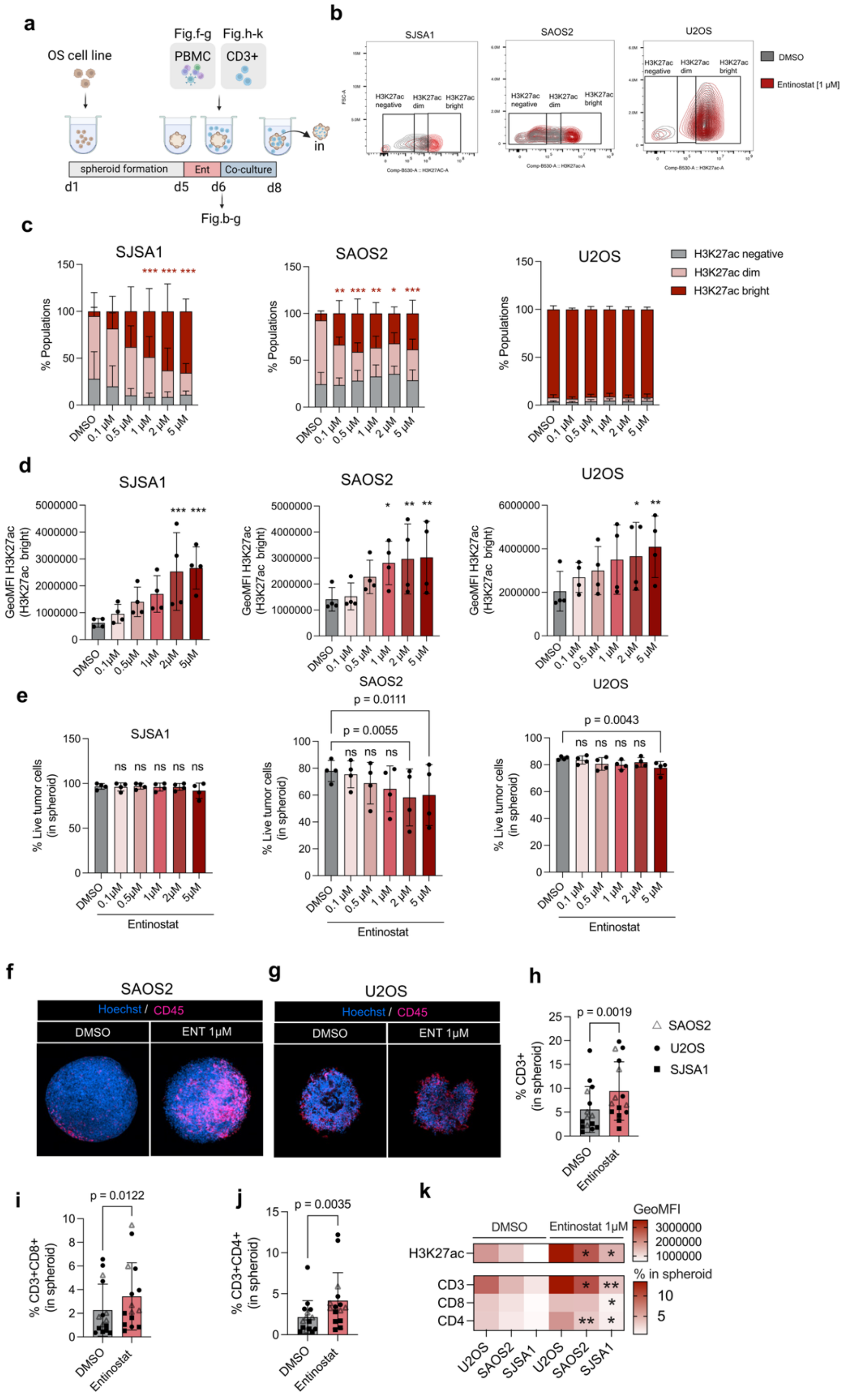

Supplementary Figure 2. Induction of H3K27ac through Entinostat augments T cell infiltration in osteosarcoma spheroids. a) Experimental design for T cell infiltration assays. b-c) Flow cytometry analysis of H3K27ac populations (negative, dim, and bright) in tumor spheroids. p-values are shown for the H3K27ac bright populations. d) Geometric mean fluorescence intensity (GeoMFI) of H3K27ac bright cells. e) Assessment of cytotoxicity. f-g) Representative images of SAOS2 and U2OS spheroids pre-treated with 1μM Entinostat or DMSO and cultured with PBMC. h-j) Infiltration of T cells in OS spheroids. Each dot represents a single independent experiment. k) Heatmap depicting H3K27ac GeoMFI of H3K27ac^{+} tumor cells in U2OS, SAOS2, and SJSA1 spheroids with DMSO or 1μM entinostat coupled with frequencies of tumor-infiltrated CD3, CD8, and CD4 T cells. Significant p-values are displayed for entinostat treatment compared to the respective DMSO control. P-values were calculated by two-way ANOVA for (b-d), one-way ANOVA for (e-g), and paired T-test for (j-m) where * = p<0.05, ** = p<0.01, and *** = p<0.001

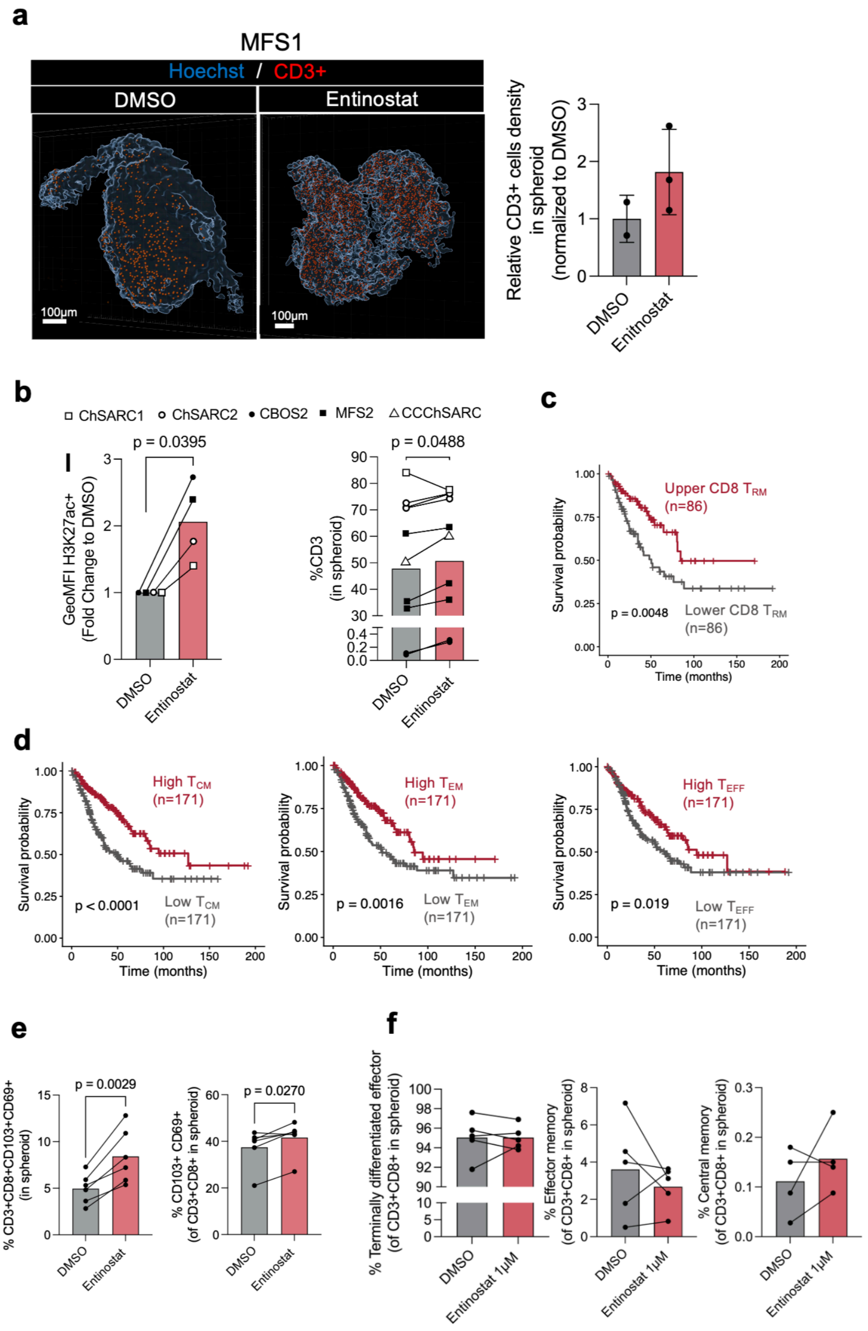

Supplementary Figure 3. H3K27ac modulation by entinostat boosts T cell infiltration. a) Confocal images of MFS1 spheroids pre-treated with entinostat and co-cultured with autologous TILs for 48 hours and quantification of $CD3^{+}$ cell density within the spheroid area. Each dot represents one spheroid. b) (Left) Fold change expression of H3K27ac upon treatment with entinostat and (Right) Frequencies of TIL infiltration in matched patient-derived sarcoma spheroids. Chondrosarcoma 1 (ChSARC1), Chondrosarcoma 2 (ChSARC2), Chondroblastic osteosarcoma 2 (CBOS2), Myxofibrosarcoma 2 (MFS2), Clear cell Chondrosarcoma (CCChSARC). Each point represents one independent experiment c-d)Kaplan-Meier curves of TCGA-SARC and TARGET-OS cohorts separating patients by c) upper and lower quartile T_{RM} CD8, (n=86) patients for each group and d) median presence of specific signatures, deconvoluting for central memory T cells (T_{CM}), effector memory T cells (T_{EM}), and effector T cells (T_{EFF}). Log-rank p-value is depicted on each graph. (n=171) patients for each group. e) Frequencies of tissue-resident memory CD8 T cells defined as CD103+CD69+ in MFS1 *ex vivo* spheroids. f) Frequencies of T cell subsets within MFS1 spheroids treated with DMSO or entinostat.

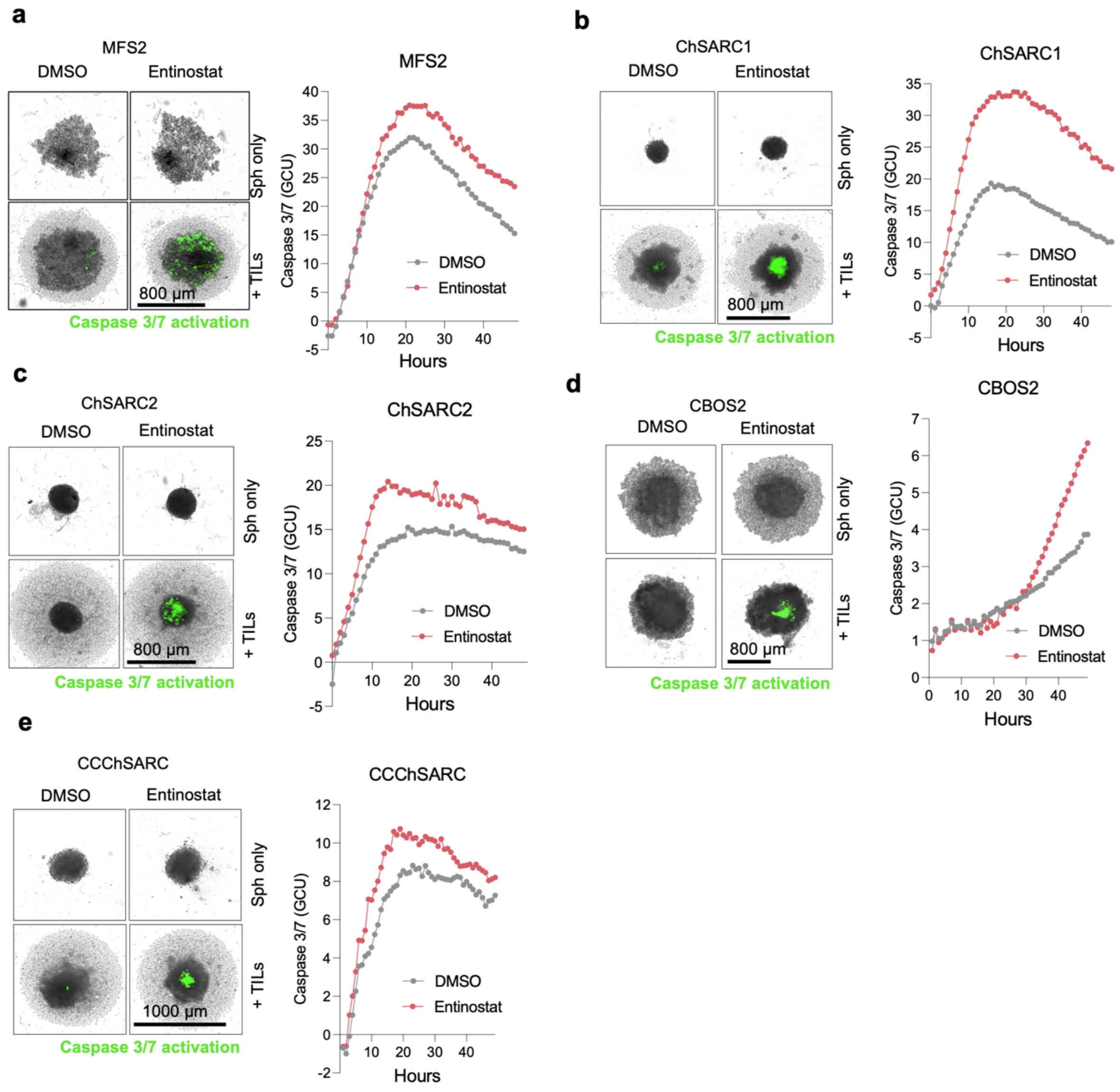

Supplementary Figure 4. Entinostat treatment enhances tumor control by autologous TILs in sarcoma patient-derived spheroids. Representative images of sarcoma spheroids at 24 hours and expression of caspase 3/7 in the presence of autologous TIL at 5:1 E:T ratio in a) Myxofibrosarcoma 2 (MFS2), b) Chondrosarcoma 1 (ChSARC1), b) Chondrosarcoma 2 (ChSARC2), d) Chondroblastic osteosarcoma 2 (CBOS2) (48 hours image), and e) Clear-cell chondrosarcoma (CCChSARC) All spheroids were normalized with their respective non-caspase and spheroid-only controls. Each experimental condition included 5-6 replicate spheroids. All patient spheroids were pre-treated for 24 hours with 1μM entinostat.

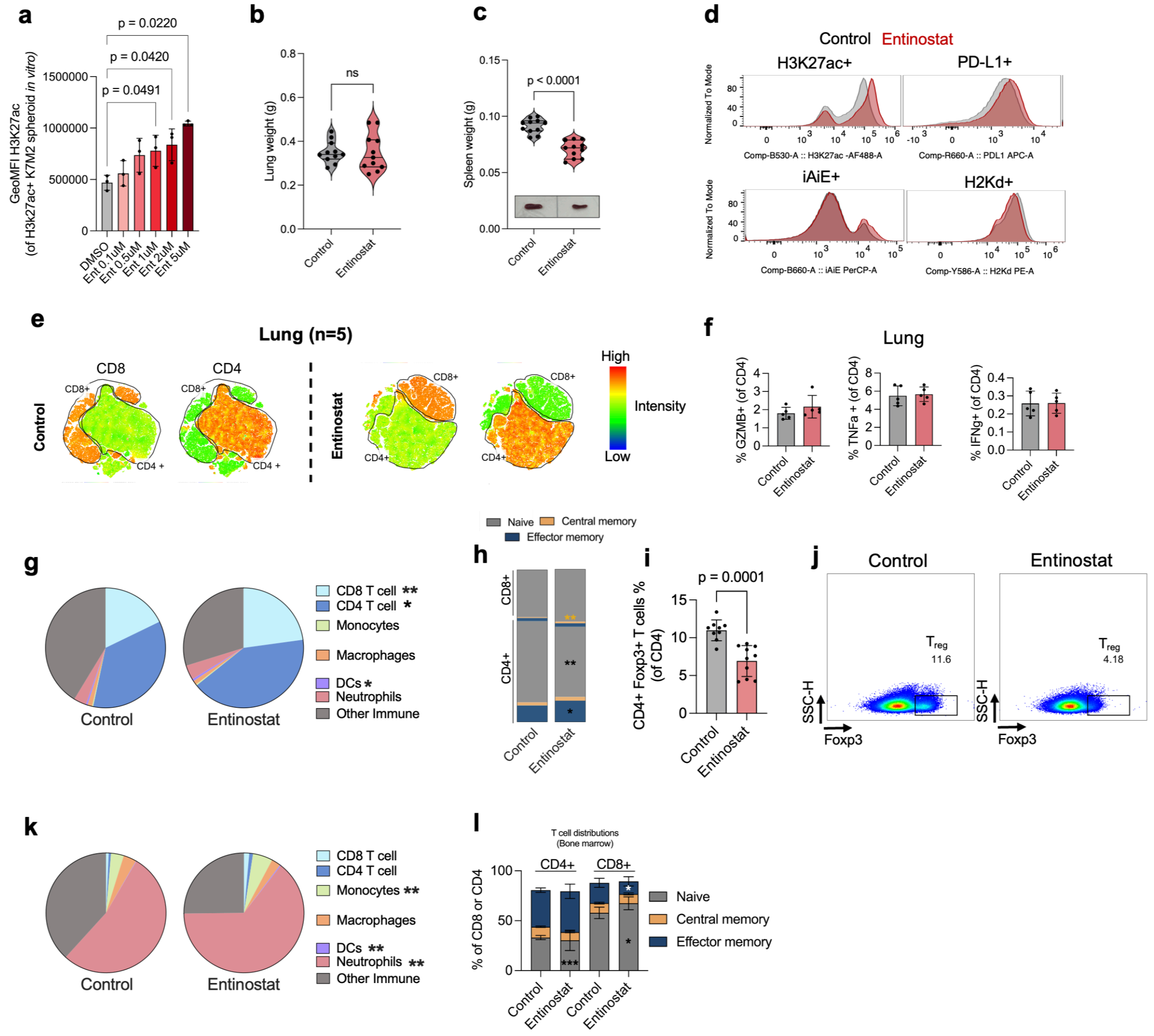

Supplementary Figure 5. *In vivo* modulation of immune populations in the spleen and bone marrow of entinostat-treated mice. a) H3K27ac levels measured as Geometric mean fluorescence intensity (GeoMFI) in H3K27ac+ K7M2 tumor cells spheroids *in vitro.* P-values were calculated by one-way ANOVA. Each dot represents one independent experimental replicate. b) Lung weights in grams (g) of resected lung from control or entinostat-treated mice on day 20. c) Spleen weight in grams (g) and representative pictures between control and entinostat mice. b-c) Each dot represents one animal. d) Representative plots of H3K27ac, PD-L1, iAiE, and H-2Kd expression in K7M2 tumor cells *in vivo*, gated as CD45-. e) t-SNE plots of six concatenated lungs per group depicting T cell separation of CD4 and CD8 subsets for Granzyme B, TNFa, and IFNg analysis (n=5 mice per group) f) Frequency of activated CD4 T cells in the lungs of mice. g) Pie-chart of immune populations changes between control and entinostat-treated spleens. h) Frequencies of memory populations in CD4 and CD8 T cells in the spleen. For spleen: n=10-11 per group. i) Frequency of Treg in spleen defined as Foxp3+ cells within the CD4+ population. j) Representative flow plot of Tregs from spleens. k) Pie-chart of immune population shifts in the bone marrow (n=5). l) Frequencies of memory populations in CD4 and CD8 T cells in the bone marrow. P-values = p<0.05 = *, p<0.01 = **, p<0.001 = ***, p<0.0001 = ****

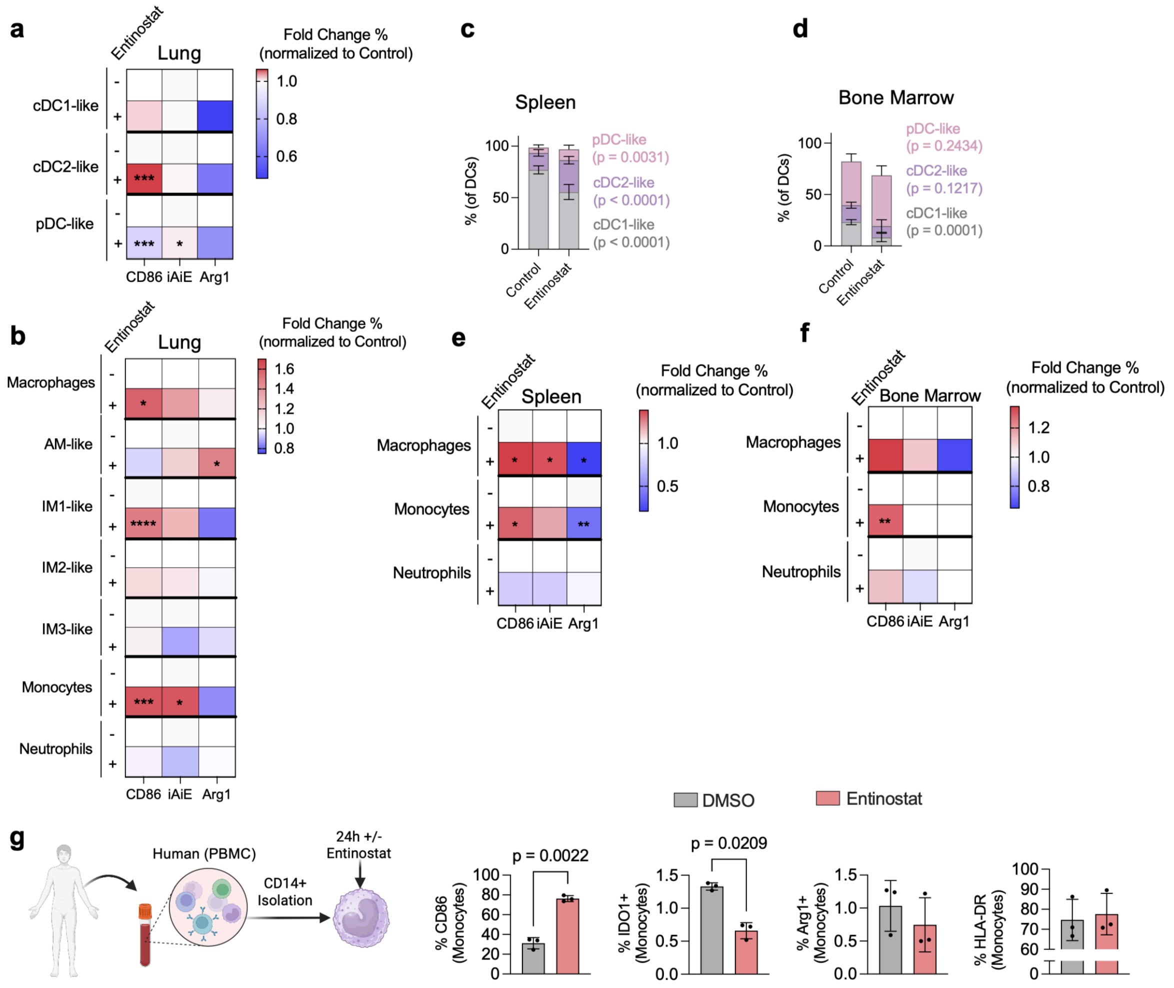

Supplementary Figure 6. Phenotype of myeloid cells *in vivo*. a) Heatmap showing fold-change (FC) frequencies of CD86, MHC class II (iAiE), and Arginase-1 positive cDC1-like cells, cDC2-like cells, and pDC-like cells in lungs of mice treated with or without entinostat. b) Heatmap showing FC of frequencies of CD86, MHC class II (iAiE), and Arginase-1 positive myeloid populations in the lungs. c-d) Frequencies of DC subsets in the spleen and bone marrow of untreated and entinostat-treated mice. e-f) Heatmap showing FC of frequencies of CD86, MHC class II (iAiE), and Arginase-1 positive myeloid populations in the spleen and bone marrow. g) Expression of CD86, IDO, Arginase-1, and HLA-DR in CD14+ cells isolated from human PBMC (n=3) treated with entinostat or DMSO for 24 hours. P-values were calculated between control and entinostat treated mice using T-tests or the Welch test were utilized to test for significance. P-values = p<0.05 = *, p<0.01 = **, p<0.001 = ***, p<0.0001 = ****

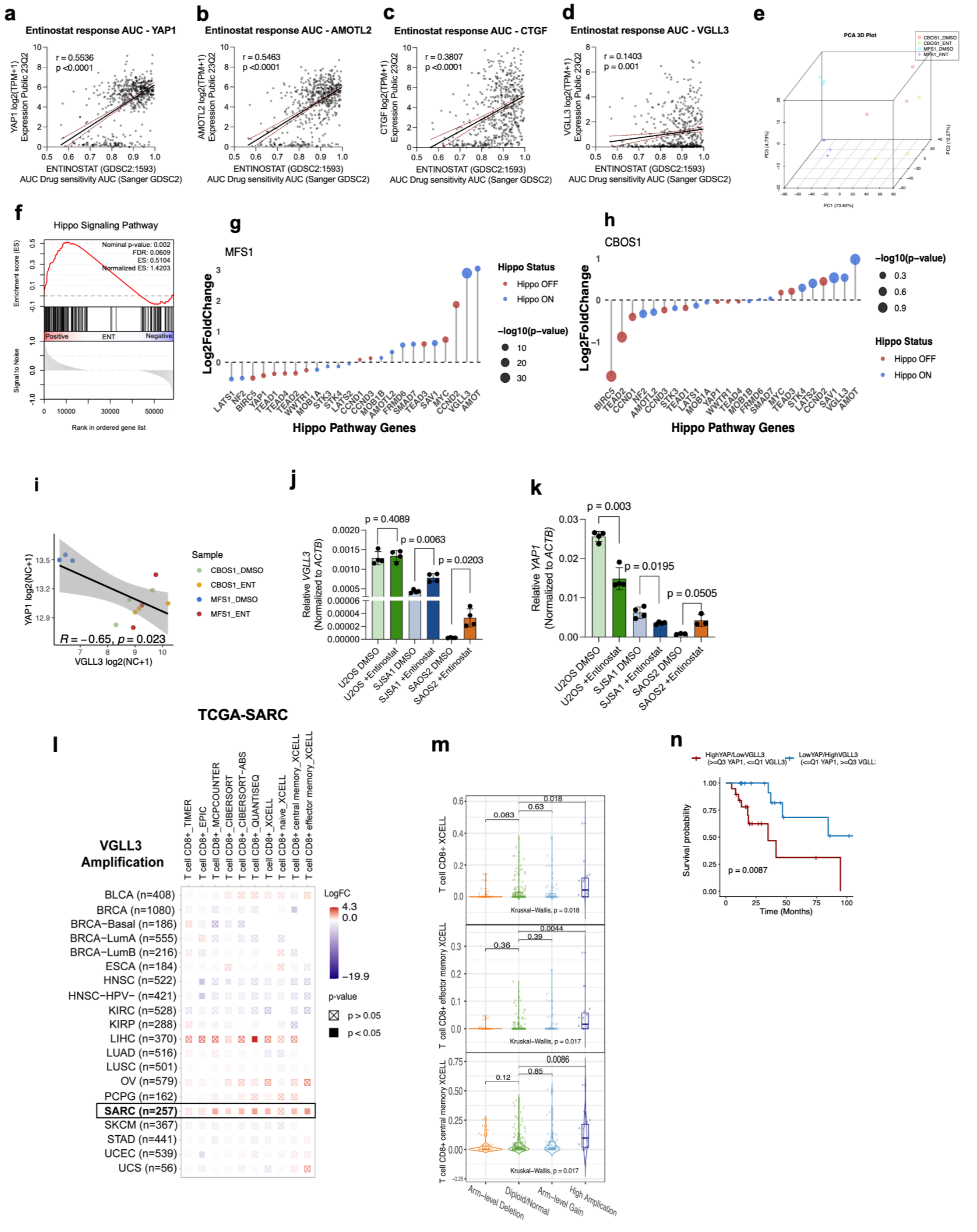

Supplementary Figure 7. VGLL3 and YAP1 are modulated by entinostat treatment. a-d) Response to entinostat across multiple cell lines from the Cell Line Encyclopedia based on the expression of a) YAP1, b) AMOTL2, c) CTGF, and d) VGLL3. e) PCA-analysis of RNAseq data. f) Gene Set Enrichment Analysis (GSEA) of KEGG pathways in the Hippo pathway upon entinostat treatment. g-h) Differential gene expression analysis in MFS1 and CBOS1 of the Hippo pathway genes, divided into on and off Hippo pathway. j-k) qRT-PCR of VGLL3 and YAP1 in tumor cells after treatment with entinostat. l) Analysis of CD8 T cell infiltration in cancers with VGLL3 amplification within the TCGA data cohort. m) Violin plot of CD8 T cell infiltrates in different VGLL3 expression groups in TCGA-SARC. n) Survival analysis in merged TCGA-SARC and TARGET-OS with YAP1^{low}/VGLL3^{high} (n=18) and YAP1^{high}/VGLL3^{low} (n=19) tumors.

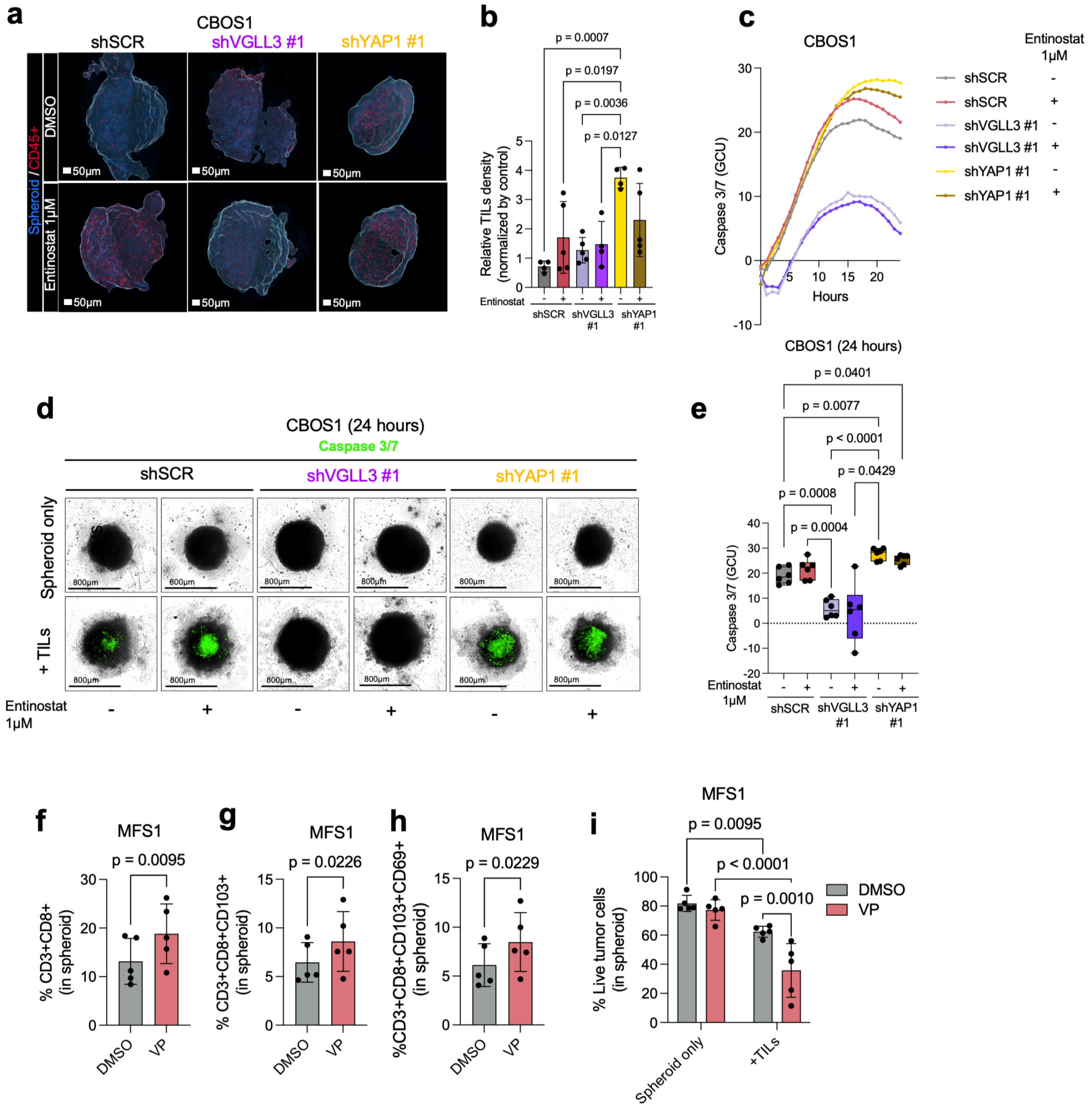

Supplementary Figure 8. YAP1 targeting enhances TIL effector functions and infiltration a) Representative images of TIL infiltration into CBOS1 spheroids. b) Relative CBOS1-TIL density normalized to shSCR DMSO control. Each dot represents one spheroid. P-values were calculated by two-way ANOVA. c) Caspase 3/7 green calibrated units (GCU) in untreated and entinostat-treated CBOS1 spheroids in the presence of autologous TIL. Values are normalized to CBOS1 tumor alone. d) Representative fluorescence images at 24 hours. e) Quantification of Caspase 3/7 in CBOS1 spheroids at 24 hours in the presence of autologous TIL. Each dot represents one spheroid/TIL culture. f) frequency of $CD8^+$ T cell infiltration into untreated or Verteporfin(VP)-treated MFS1 spheroids. g-h) Frequency of T_{RM} T cell infiltration into untreated or Verteporfin-treated MFS1 spheroids. i) Live tumors cells in the presence or absence of TIL in untreated or Verteporfin-treated (0.1ug/ml) MFS1 spheroids.

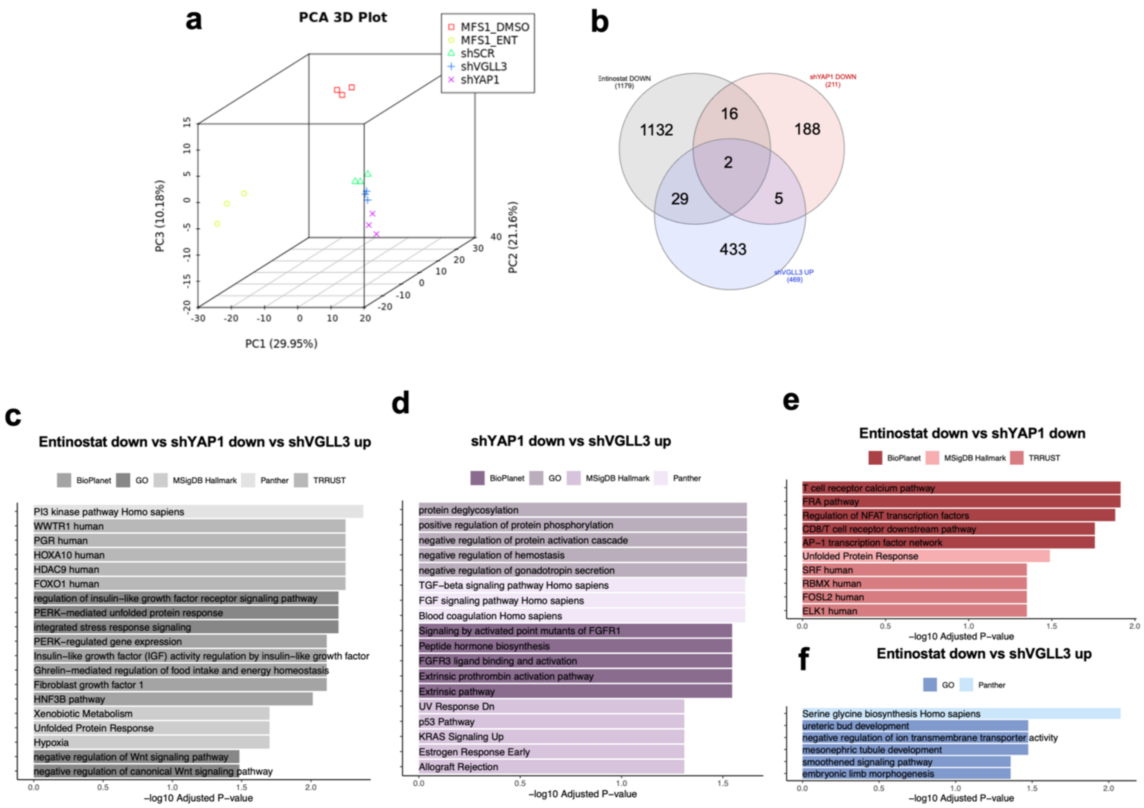

Supplementary Figure 9. Entinostat, YAP, and VGLL3 share common transcriptional pathways a) PC-analysis of RNAseq data. b) Identification of shared transcriptional modulation by intersecting differentially downregulated genes in entinostat, shYAP1, while differentially upregulated in shVGLL3. Three replicates are included in each experimental condition. c-f) Pathway enrichment analysis of intercepted DEGs in different samples.

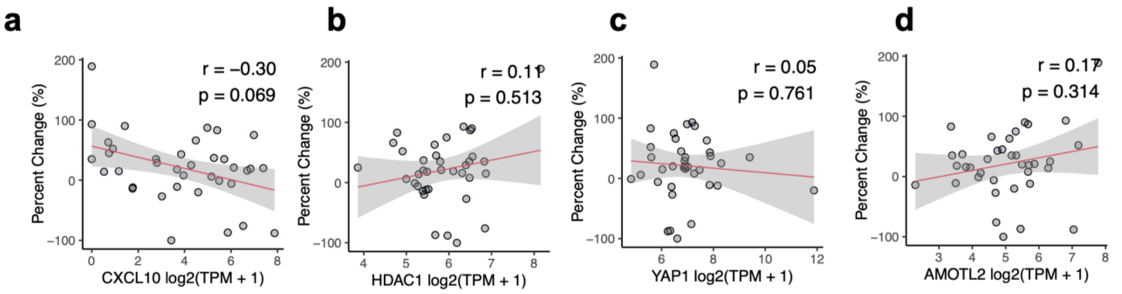

Supplementary Figure 10. a-d) Spearman correlation of gene expression log2(TPM+1) against % change in tumor size in 37 sarcoma patients treated with Nivolumab/Ipilimumab.

Supplementary Methods Figures

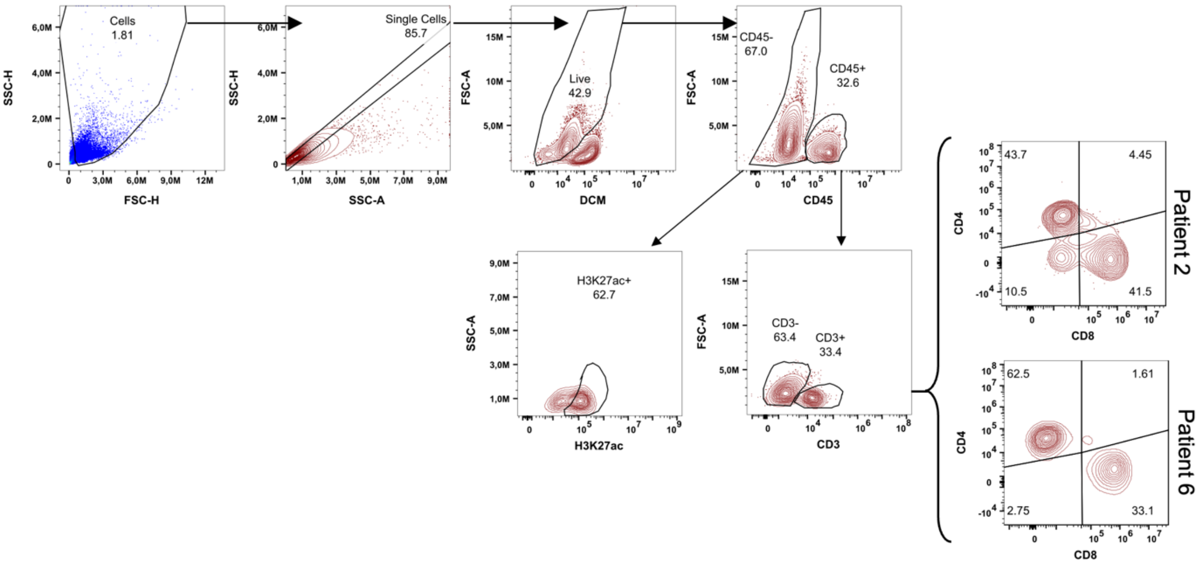

Supplementary Methods 1. Gating strategy for H3K27ac and T cells in sarcoma surgical resections.

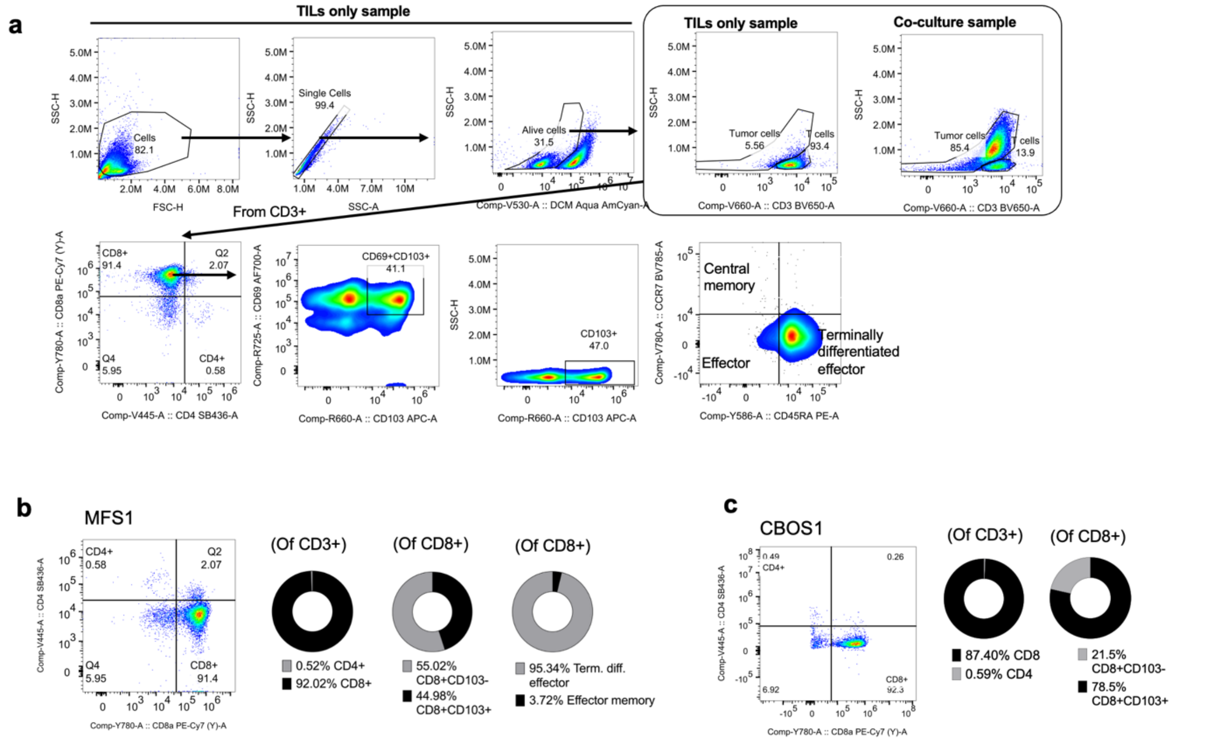

Supplementary Methods 2. a) Gating strategy for T_{RM}, T_{CM}, T_{EF}, T_{EMRA} and T_{N} in patient-derived TIL and spheroid co-cultures. b) TIL phenotype of MFS1 patient. c) TIL phenotype of CBOS1 patient.

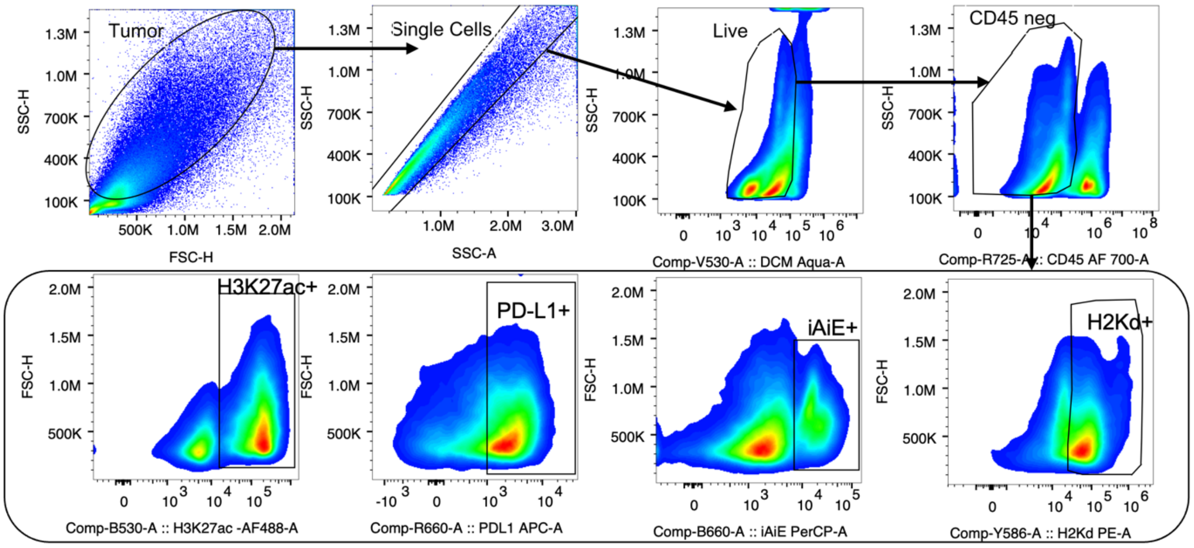

Supplementary Methods 3. Gating strategy for K7M2 tumor cells in vivo.

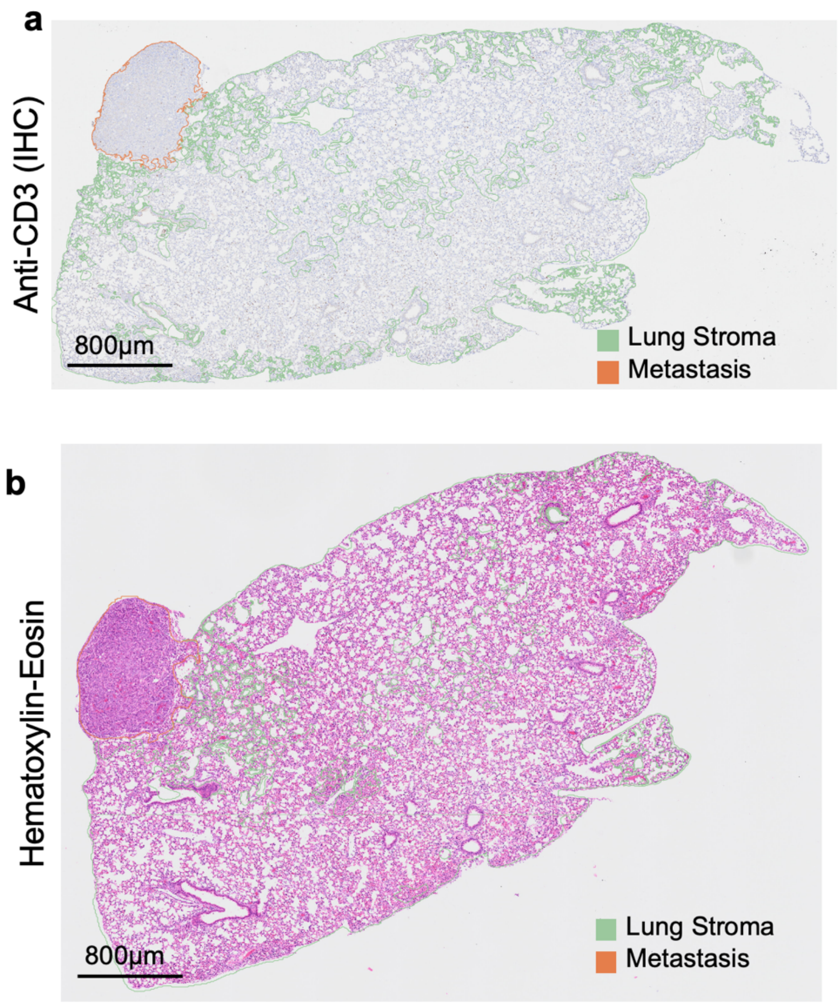

Supplementary Methods 4. Representative image of **a)** Immunohistochemistry for CD3 and b) Hematoxylin-Eosin of K7M2 tumors. Delineation of metastasis and stroma was performed by QuPath software.

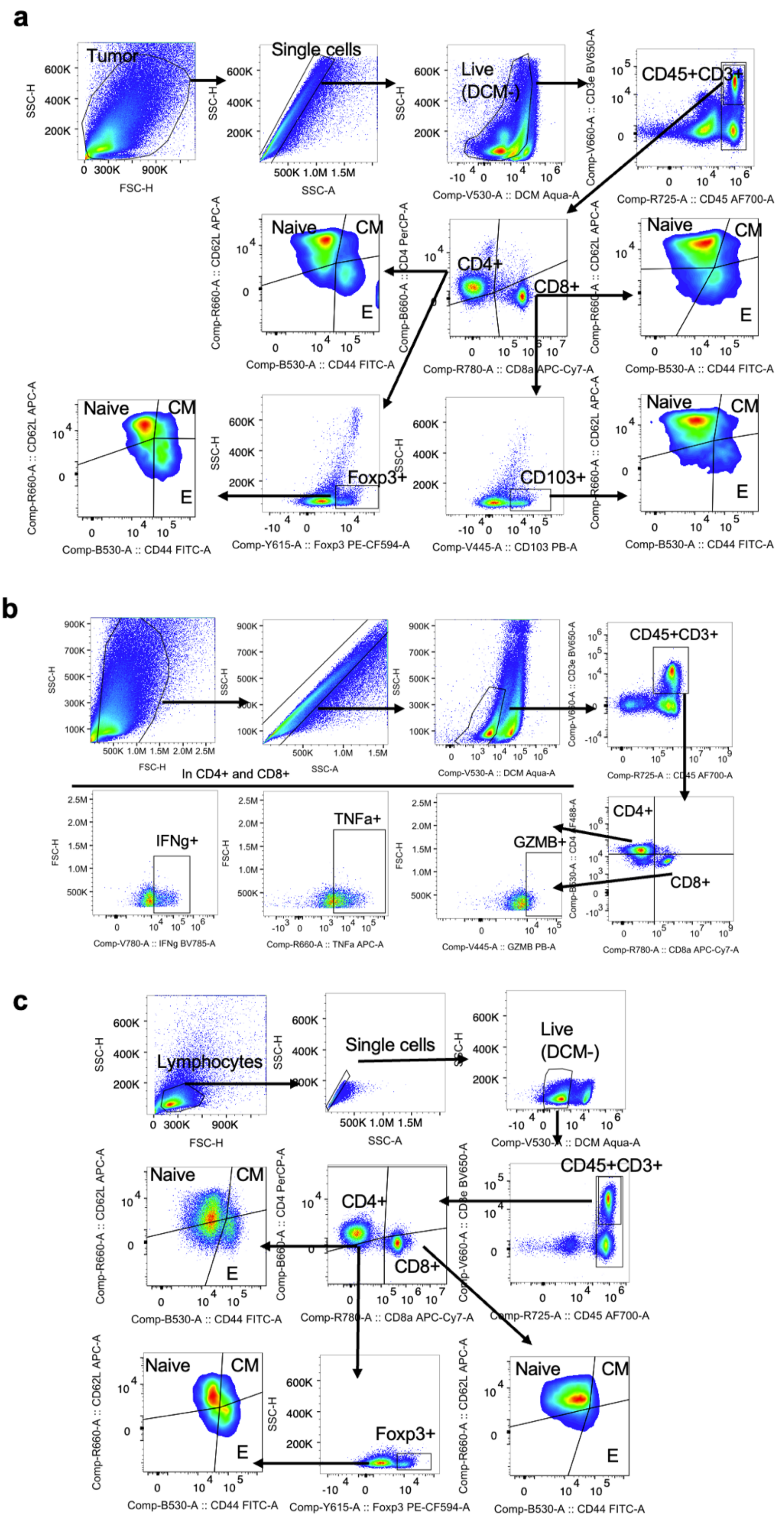

Supplementary Methods 5. Gating strategies for a) T cell phenotyping in lungs, b) for T cell cytokines, and c) T cell phenotype in spleen, and bone marrow.

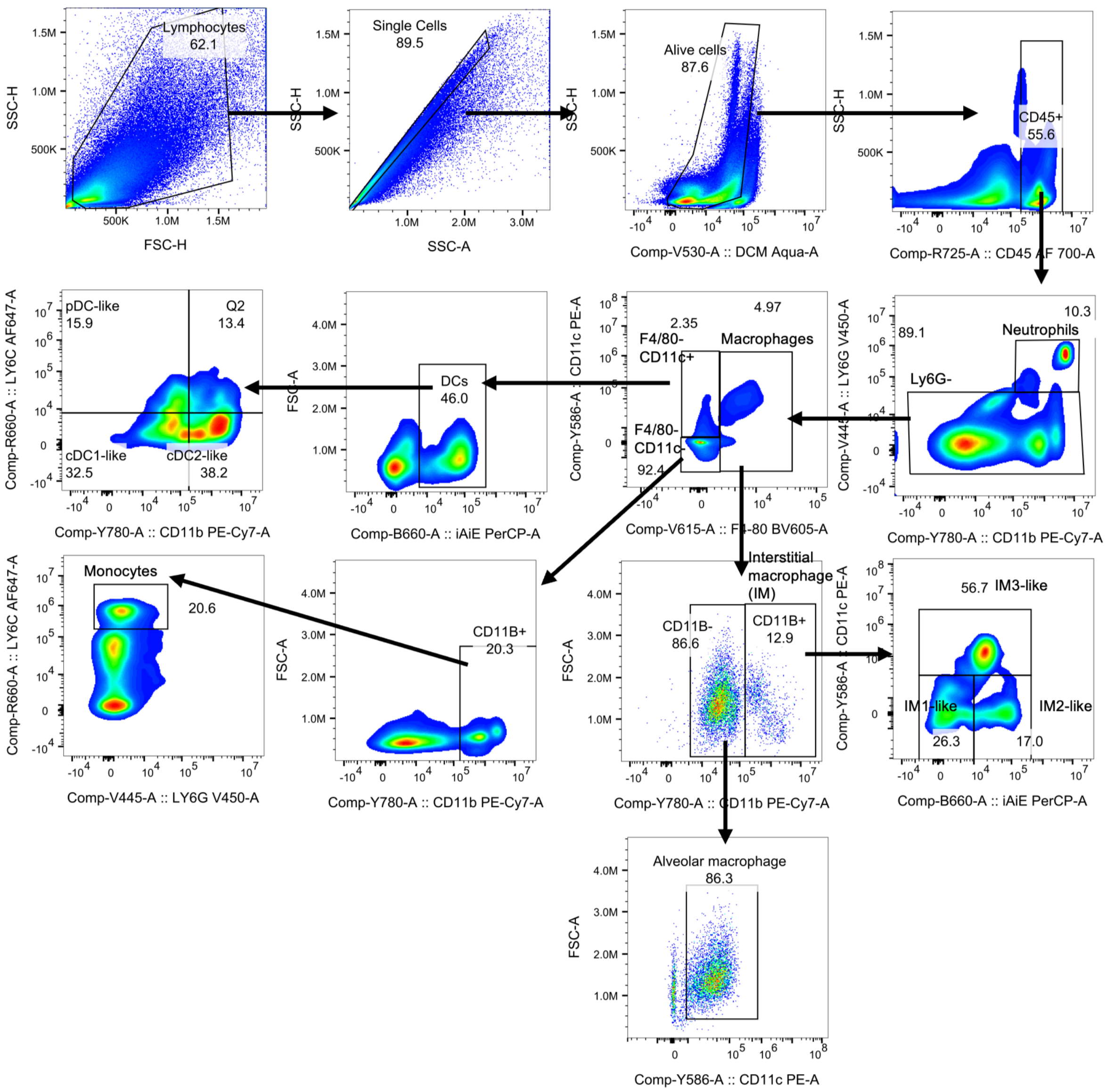

Supplementary Methods 6. Gating strategy for myeloid cells.

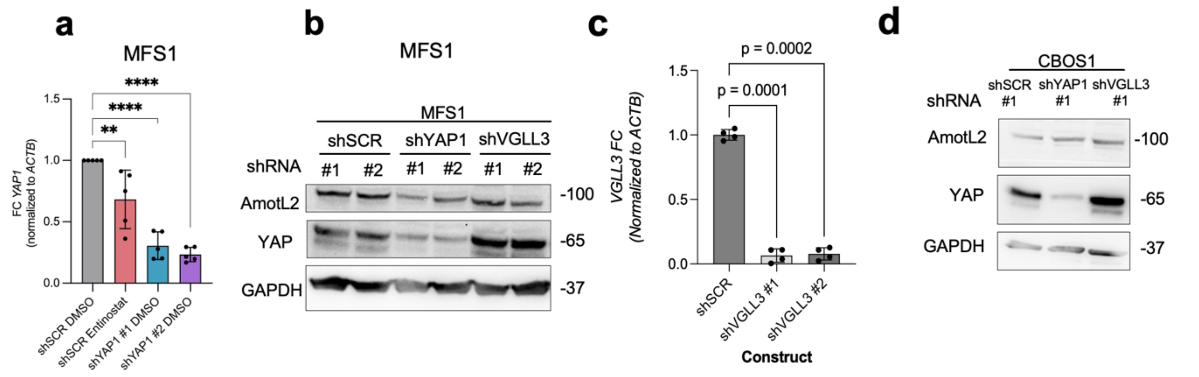

Supplementary Methods 7. Knock-down of shYAP1#1 and shYAP1#2 construct at transcriptome level in MFS1 tumor cells. b) Western blot for YAP1 and AMOTL2 for shYAP1 and shVGLL3. c) Expression of VGLL3 in shVGLL3 constructs #1 and #2 in MFS1 tumor cells. d) Protein validation of YAP1 knock-down of construct #1 in CBOS1.

Supplementary Tables

Number patient	Sex	Diagnosis
1	F	Chondroblastic Osteosarcoma
2	F	Leiomyosarcoma
3	M	Myxofibrosarcoma
4	F	Mesonephric-like carcinosarcoma
5	M	MPNST
6	M	Liposarcoma
7	M	Undifferentiated Pleomorphic Sarcoma
8	M	Dedifferentiated liposarcoma

Supplementary Table 1. Patient characteristics for immune phenotyping

Number patient	Sex	Pseudoname	Diagnosis
9	M	CBOS1	Chondroblastic osteosarcoma
10	M	MFS1	Grade 3 Myxofibrosarcoma
1	F	CBOS2	Chondroblastic osteosarcoma
3	M	MFS2	Myxofibrosarcoma
11	M	ChSARC1	Chondrosarcoma
12	F	ChSARC2	Chondrosarcoma
13	M	CCChSARC	Clear-cell chondrosarcoma

Supplementary Table 2. Patient characteristics of primary cell cultures TILs and tumors

Supplementary Methods

Sarcoma tissue processing and tumor cell culture

Tumor samples were fragmented into 2mm pieces following chemical and mechanical dissociation using the Tumor Dissociation kit (130-095-929, Miltenyi) and gentleMACS Octo dissociator (130-096-427, Miltenyi). Part of the single cell suspensions were stained by flow cytometry to evaluate immune infiltration and expression of H3K27ac. To isolate tumor cells, the human Tumor Cell isolation kit (130-108-339, Miltenyi) was used following the manufacturer's instructions. For culture of primary tumor cells, negative-selected sarcoma cells were cultured in DMEM medium supplemented with Glutamax-I (Gibco, 61965-026), 1% penicillin/streptomycin (Gibco, 15140-122), 10% heat-inactivated Foetal Bovine Serum (FBS) (Gibco, A5256701), 1x Sodium Pyruvate (Gibco, 11360-039), and 1x Primocin (InvivoGen, NC9392943). Primary cells were cultured at 37ºC in a humidified incubator with 5% CO_2 for at least 10 passages and used for experiments until a maximum of 25 passages.

T cell expansion

For isolation and expansion of TIL, single cell suspensions and positive selected cells from the Human Tumor cell isolation kit (130-108-339, Miltenyi) were cultured in TIL medium (47% X-VIVO 20 (Lonza, 04-448Q), 47% AIM V medium (1x) (Gibco, 12055-091), 1% penicillin/streptomycin, 5% hAB serum (Karolinska Hospital), containing 1000 IU/mL IL-15 (PreproTech, AF-200-15-100UG) for 1 week at 37ºC in a humified incubator with 5% CO_2. Following microscopic confirmation of colony formation, TIL were harvested, counted, and processed for rapid expansion protocol (REP) as previously described [1] with 40 Gy irradiated feeder cells from at least four different healthy donor PBMC. Ultra-LEAF™ Purified anti-human CD3 Antibody - OKT3 (30ng/mL, Biolegend 317326) and 500IU/mL IL15 in AIM V medium, 2 % hAB serum. Cells were kept in culture at 37ºC in a humidified incubator with 5% CO_2. Fresh TIL media supplemented with 500 IU/mL IL-15 was added on days 5, 9, and 12. After 20 days, TIL were harvested and used for experiments.

Lentiviral-induced knock-down of patient-derived *ex vivo* sarcoma cultures

Lentiviruses were generated by transfection with Lipofectamine 3000 (Invitrogen, L3000001) of HEK293T cells with third-generation packaging plasmids (Addgene, #12253, #12251, #8454) and either scrambled control short hairpin RNA (shRNA SCR) (shRNA scrambled control; SHC016; Sigma), shRNA YAP#1 (Addgene, #42540), shRNA YAP#2 (Addgene #42541), shVGLL3#1 (Vector builder, VB231101-1341xmh), or shVGLL3#2 (Vector Builder, VB231105-1031aaq). Lentivirus-containing supernatant was subsequently used to transduce patient-derived cells in a 1:1 dilution with DMEM supplemented with 10% FBS, 1x Sodium Pyruvate, and 5μg/mL Polybrene Infection/Transfection Reagent (Sigma, TR-1003-G), and were kept in culture overnight. Selection was performed with 2μg/mL puromycin (Gibco, A1113803) prior to downstream studies.

Real-time killing assay analysis

In every experiment, 30 spheroids were randomly selected as training images at different time points. Values of sensitivity and background were then adjusted to specifically detect the spheroid area (Usually 90-100% sensitivity). Minimum size values of 3x10^5m2 were applied to avoid incorrectly detecting debris. After visual confirmation of correct spheroid detection, analysis was applied to all experimental pictures. Upon analysis termination, GCU (Green Fluorescent mean intensity) values, consisting of the mean green fluorescence per pixel within the spheroid, were obtained per spheroid and timepoint. For the calculation of TIL-mediated killing, the mean of spheroid-only GCU value was calculated and subtracted from co-culture GCU to obtain specific TIL-mediated Caspase 3/7 activation. Evaluation of viability and GCU was performed in all experiments for TILs and spheroids-only cultures. Spheroid images were randomly selected and retrieved using Incucyte software to ensure representative visualization. Background-noise green fluorescence-subtracted images adjusted based on the spheroid-only condition were obtained from the software for standardized image display.

Murine osteosarcoma model and tissue processing

K7M2 cells were thawed and expanded in complete DMEM GlutaMax. Mycoplasma test was performed before cell injection by using PCR testing through Eurofins Genomics. Mice were acclimatized for 2 weeks before starting the experiment, and cage enrichment and animal welfare were ensured throughout the experiment. Mice were randomly assigned to each treatment group.

Prior to the mice being sacrificed, organ isolation was performed. The tissues were kept in PBS until further processing. Lungs were mechanically fragmented and chemically processed to single cell suspension following the Miltenyi Lung Dissociation Kit (Miltenyi Biotec, 130-095-927). Spleen was placed mechanically disaggregated in a cell strainer (70μm) while continuously adding PBS, ensuring maximum cell recovery. Both femurs were collected by eliminating excess tissue. Bone epiphyses were removed, and PBS with 10%FBS was flushed through. The filtered cell suspensions were washed and centrifuged (300g, 5 minutes). Single-cell suspensions for lungs, spleen, and bone marrow were incubated with 1x ACK Buffer (Gibco, A1049201) at 37ºC for 5 minutes for red-blood cell removal. To stop the reaction, PBS was added, followed by centrifugation (300g, 5 minutes, RT). Finally, the pellet was resuspended in DMEM-supplemented medium, and cell concentration and viability were determined. Single-cell suspensions were used to define the tumor phenotype, the myeloid compartment subpopulations, and the T cell phenotype and effector activity by flow cytometry. Samples were excluded from flow cytometry analysis in the event of cell viability below 10%.

Immunohistochemistry staining and analysis of murine lung tissue

Hematoxylin and eosin staining were used alongside to accurately identify metastatic lesions, which were then analyzed in QuPath (Supplementary Methods Fig.4). Lungs were collected and washed with PBS and fixed with ice-cold 4% Paraformaldehyde for 48 hours at 4 °C, followed by dehydration with increasing concentrations of ethanol for embedding in paraffin (FFPE). 5-μm sections were used for immunohistochemical staining with anti-CD3 (rabbit, Dako Agilent, A0452) at a 1:2000 dilution. Tissues were imaged using an Olympus VS200 whole scan brightfield acquisition system.

QuPath Software was used to analyze T cell infiltration. The metastatic tumor areas were manually annotated, aided by paired hematoxylin-eosin staining and a specialized pathologist. The rest of the surrounding tissue, consisting of the healthy lung, was annotated as stroma. Batch correction was applied by using the same stain vector settings across all images to normalize DAB intensity. The positive cell detection module was used to automatically detect DAB-positive cells comprising the $CD3^+$ population. Detection thresholds for $CD3^+$ cells were established using non-tumor-bearing lung controls for each experimental repeat using DAB mean optical density. This approach minimized inter-experimental variability and allowed for accurate $CD3^+$ cell infiltration scoring quantification. The frequency of $CD3^+$ cells from the total of cells and $CD3^+$ cells/mm^2 were obtained for each annotated area.

Flow cytometry analysis

Single cells were stained with viability dye 1:1000 LIVE/DEAD™ Fixable Aqua Dead Cell Stain Kit, (Invitrogen, L34966A) or Fixable Near-IR Dead Cell Stain Kit (Invitrogen, L10119) in PBS 15 minutes at room temperature (RT). After FACS buffer washes (PBS, 2%FBS, and 2mM EDTA), surface antibodies in FACS buffer were incubated for 30 minutes at 4ºC. Cells were then washed and prepared for intracellular and intranuclear staining. For that, cells were permeabilized by adding Fixation/Permeabilization solution (1:4 Fixation/Permeabilization Concentrate (Invitrogen, 00-5123-43) and 3:4 Perm Diluent (Invitrogen, 00-5223-56)) for 30 minutes at 4ºC in the dark. Next, 1x Permeabilization Buffer (Invitrogen, 00-8333-56) was used to wash, and the intracellular antibody mix was added and incubated for 30 minutes at RT.

For flow cytometry staining of single cell suspensions from sarcoma patient resections, after viability staining, the samples were incubated for 5 minutes with Human TruStain FcX™ (Fc Receptor Blocking Solution) (Biolegend, 422302).

For cytokine production assessment of murine tissues, 1.5 million cells per sample were seeded in a 24-well flat-bottom plate and treated with 1:1000 Golgi Plug (BD, 51-2301KZ) and 1:1500 Golgi Stop (BD, 51-2092KZ) and kept in culture for four hours at 37ºC in a humidified incubator with 5% CO_2. Single-cell suspensions were then washed with FACS buffer, and centrifuged (500g, 4

minutes, RT) and blocked with 1:10 mouse FcR blockade (for murine cells) (Miltenyi Biotec, 130-092-575) in MACS buffer (PBS with 5% BSA and 2mM EDTA) for 10 minutes at 4ºC.

Flow cytometry data was aqcuired on a Novocyte Quanteon flow cytometer (ACEA Biosciences). Antibodies and dilutions are listed in **Supplementary Materials Table 1**. FCS files were analyzed using FlowJo (Version 10.10.0). Single stain compensation control beads (Thermo Fisher Scientific, A10497, and A10346) were used following manufacturer's instructions. Compensation matrices were generated and applied to the samples. tSNE plots for cytokine activity were generated as follows: compensated flow cytometry files (FCS) were gated on live, single cells, $CD45^{+}CD3^{+}$, and further concatenated for tSNE generation using tSNE plug-in in FlowJo. The markers for tSNE generation included GZMB, IFNg, TNFa, CD4, and CD8. Heatmaps for the expression of Arg1, iAiE, and CD86 in myeloid populations represent fold changes from the frequency average of each positive population in the control mouse group. In that case, the p-value was calculated from the original raw frequency differences. Gating strategies are displayed in Supplementary Methods Figure 1-3, 5 and 6. Description of markers for definition of immune populations are detailed in **Supplementary Materials Table 2.**

Assessment of infiltration using 3D confocal imaging

Spheroids were washed in PBS to eliminate non-infiltrating cells and fixed for 30 minutes with ice-cold 4% PFA (Invitrogen, FB002). This was followed by 40 minutes permeabilization (0.5% Triton-X (Sigma, T8787) in PBS), 1 hour blocking (0.1% Triton-X and 2.5% BSA (Sigma, A9647) in PBS), and incubation with primary antibody; anti-CD45 (Abcam, ab8216,1:300) or anti-CD3 (Abcam, ab135372, 1:50) overnight in blocking buffer at 4°C. The following day, after primary antibody washes with 0.1% Triton-X in PBS, secondary antibodies Donkey anti-Mouse IgG (H+L) Highly Cross-Adsorbed Secondary Antibody, Alexa Fluor™ 647 (Invitrogen, A-31571) or Donkey anti-Rabbit IgG (H+L) Highly Cross-Adsorbed Secondary Antibody, Alexa Fluor™ 555 (Invitrogen, A-31572) at 1:500 concentration were incubated overnight at 4°C together with 1:2000 Hoechst 33342 Trihydrochloride (Stock 10mg/mL) (Invitrogen, H1399). Subsequent clearing was performed with OmniPaque (Iohexol 350 mg L/mL, GE Healthcare) overnight at room temperature and transferred to a μ-Slide 18 Well (e.g., Ibidi, 81816) for visualization. As controls of unspecific binding

and specificity of antibodies, spheroids without target cells were fully stained to ensure non-specific primary antibody bindings, and spheroids with target cells were stained with secondary antibody only to identify unspecific binding.

Image acquisition was performed in a LSM800 confocal microscope (Zeiss Axio Observer Z1/7) using ZEN Imaging Software (Version 2.6). Hoechst staining was used to define spheroid volume and depth of Z-Stack acquisition as described previously [2]. Converted files in Imaris File Converter (Version 10.1) were analyzed in Imaris Analysis Software (Version 10.1), as described[2]. Hoechst channel was used to create the 3D spheroid surface. Spheroid surface was further used to mask the lymphocyte channel consisting of CD3 or CD45 antibody through MatLab Channel Arithmetic image processing within Imaris. Spots were further defined using the newly created channel, consisting of the infiltrated lymphocytes inside the spheroid surface, and statistics were retrieved for the volume of the spheroid surface and the number of lymphocytes. Parameters for spheroid area and lymphocyte detection were stored and used for batch analysis of experimental replicates to keep conditions consistent.

Chromatin Immunoprecipitation

Chromatin Immunoprecipitation was performed using the iDeal ChIP-seq kit for transcription factors (Diagenode, C01010055) according to the manufacturer's instructions with modifications. MFS1 cells were treated with entinostat or DMSO for 24 hours and cross-linked with 1% formaldehyde at RT for 15 minutes with gentle shaking. The cross-linking was quenched with 0.125 M glycine, and the cells were washed once with ice-cold 1× PBS keeping cells on ice. After lysed with lysis buffer iL1b and iL2, the chromatin was sheared by sonication (Branson Digital Sonifier) for 14 minutes with 30 sec on and 30 sec off cycles. For immunoprecipitation, ChIP grade antibodies were incubated with prewashed protein A–coated magnetic beads for 4 hours at 4 °C under rotation. Sheared chromatin was then added to beads and incubated overnight at 4 °C under rotation. Beads were spun and washed according to the protocol. Immunoprecipitated and input DNA were then eluted and purified according to the manufacturer's protocol. Real-time PCR was performed using 2% of immunoprecipitated or input DNA per reaction using SYBR green reagents according to the manufacturer's protocol. Fold enrichment (2−DDCT) was calculated using input controls and IgG

controls as background. Antibodies used for ChIP experiments were as follows: Rabbit Polyclonal H3 acetyl K27 ChIP grade antibody (ab4729; Abcam), Rabbit IgG (C15410206; Diagenode), Mouse RNA Polymerase II antibody (39097; Active Motif), Mouse IgG (I8765; Sigma Aldrich).

Western Blot

Protein expression was analyzed using precast Bis-Tris-PAGE (Invitrogen). Cells were washed in 1× PBS before being lysed in 1× RIPA lysis buffer (EMD Millipore Corporation). Cell lysates were boiled at 100 °C for 5 minutes and mixed with the NuPAGE LDS Sample Buffer (4×; Invitrogen), the NuPAGE Sample Reducing Agent 10× (Invitrogen), and total protein separated on a NuPAGE 4% to 12%, Bis-Tris, polyacrylamide gradient gel (Invitrogen) before Western blotting on Pure Nitorcellulose 0.2- μm membranes (PerkinElmer). Membranes were probed with primary antibodies, followed by Amersham ECL anti-mouse (NA931-1ML; CiteAb) or anti-rabbit antibodies (NA934-1ML; CiteAb) for detection of probed proteins using enhanced chemiluminescence (Perkin Elmer). Chemiluminescence was detected using an iBright FL1500 (ThermoFisherScientific) imager. Antibodies used for Western blot: rabbit pAb anti-AmotL2 (Innovagen). Anti-YAP rabbit mAb (#14074; Cell signaling Technologies), Mouse anti-GAPDH (ab181602, Abcam).

RNA isolation

After the treatment, at least 24 spheroids per condition were collected in Eppendorf tubes, washed twice with 1x PBS (Gibco, 14190-144), and centrifuged (500g, 4 minutes). Spheroid lysis and RNA isolation followed the manufacturer's instructions from PureLink RNA Mini Kit (Invitrogen, 12183018A) combined with on-column PureLink DNase kit (Invitrogen, 12185010). RNA yield and quality were defined by absorbance at 260 and 280nm using NanoDrop 1000 spectrophotometer (NanoDrop Technologies).

Quantitative Real-Time PCR and analysis

cDNA was generated ensuing manufacturer's indications from the SuperScript IV Reverse transcriptase manufacturer protocol (Invitrogen, 18090050), together with Oligo d(T)$_{20}$ primer

(Invitrogen, 58862), dNTP mix (Invitrogen, R0192), SSIV Buffer RT Reaction Buffer (Invitrogen, 18090050B), DTT (Invitrogen, 707265ML) and RNAseOUT Recombinant RNase Inhibitor (Invitrogen, 10777-019) in a C1000 Touch Thermal Cycler (BIO-RAD). The qPCR was performed in duplicates using PowerUP SYBR green Master Mix and following manufacturer's instructions (Thermo Fisher Scientific, 4367659). 20ng of cDNA were used as input and 400nM of each corresponding reverse and forward primers (**Supplementary Materials Table 3**) in MicroAmp Optical 96-well Reaction Plate (Thermo Fisher Scientific, N8010560). The assay was performed in QuantStudio 7 Flex qPCR system (Thermo Fisher Scientific). qRT-PCR data were analyzed using two approaches: For treated versus untreated conditions and most of the comparisons relative fold-change was determined using the ΔΔCt method, where ΔCt was calculated as (Ct_{target} - Ct_{ACTB}), and fold-change was expressed as $2^{-\Delta\Delta Ct}$ relative to the untreated control. However, uniquely to compare gene expression of YAP1 and VGLL3 across different cell lines the 2^{-Ct} method was used, with normalized expression calculated by dividing the 2^{-Ct} value of the target gene by that of the housekeeping gene (ACTB) in each sample. These approaches allowed for accurate cross-cell-line comparisons and fold-change quantification.

Confocal microscopy and analysis of YAP1

Patient-derived cells were seeded on µ-Slide 8 well (Ibidi) and treated 24 hours later with 1 µM entinostat or vehicle (DMSO) for one day. Subsequently, cells were washed with PBS and fixed with Image-IT 4% Paraformaldehyde (Invitrogen, I28800) for 15 min at 4°C. Cells were subsequently permeabilized with 0.4% Triton-X in PBS for 20 min. Washes were conducted throughout in TBS-Tween 0.1%. Blocking was conducted with 2% BSA in 0.1% PBS Triton-X for YAP1 staining. Thereafter, primary antibody was added overnight at 4°C. The secondary antibody was incubated for 1h at a 1:500 dilution in the blocking buffer, followed by nuclei counterstain with Hoechst 1:1000. Confocal images were acquired in a LSM800 confocal microscope (Zeiss Axio Observer Z1/7) using ZEN Imaging Software (Version 2.6). Confocal image analysis was performed using CellProfiler v5.0 (www.cellprofiler.org). Images were imported, and metadata were extracted from file names and folder structure. Channels were assigned to the respective detection: DNA or YAP1. Nuclei were segmented from the DNA channel, using primary object identification with a

typical diameter range of 80–300 pixels. Whole-cell boundaries were defined by propagating outward from the nuclei using the YAP1 channel, depending on the staining performed. Cytoplasmic regions were generated by subtracting the nuclear area from the whole-cell masks. Integrated intensity measurements were extracted for each compartment. Nuclear-to-cytoplasmic YAP1 intensity ratios were calculated.

Public patient cohort epigenome score analysis

Publicly available RNA-seq profiles from the Therapeutically Applicable Research to Generate Effective Treatments – Osteosarcoma (TARGET-OS, n=84) program and the Genotype-Tissue Expression (GTEx) project (skeletal muscle controls, n = 396) were analyzed. Gene models and biotypes followed GENCODE v32. For each cohort, gene-level quantifications were harmonized to Ensembl gene IDs, mapped to HGNC symbols, and collapsed by symbol (mean TPM when multiple Ensembl IDs mapped to the same symbol). Expression values were transformed as log2(TPM + 1). To mitigate cross-study effects, batch effects were removed by cohort (TARGET vs GTEx) using limma::removeBatchEffect, followed by limma::normalizeBetweenArrays. Principal component analysis (PCA) on centered and scaled matrices was used for quality assessment before and after correction and to visualize tumor vs control separation. Where indicated, differential expression between TARGET and GTEx was performed with limma and Benjamini-Hochberg false discovery rate control (FDR < 0.05).

Histone-mark annotation and tumor-specific epi-PCG gene sets

Genome-wide peak sets for six histone modifications (H3K27ac, H3K4me1, H3K4me3, H3K36me3, H3K27me3, H3K9me3) were downloaded from ENCODE for the SJSA1 osteosarcoma cell line and for osteoblast cell lines (controls). Using ENCODE-provided peaks, peaks to genes were mapped with the ChIPseeker R package on hg38, leveraging the Bioconductor packages TxDb.Hsapiens.UCSC.hg38.knownGene and org.Hs.eg.db. Promoter association was defined as overlap within -2000 bp to +500 bp of the transcription start site using the ChIPseeker R package). Enhancer annotations were taken from the public FANTOM5 permissive enhancer atlas. Enhancer-

to-gene assignments followed the corresponding FANTOM5 enhancer–promoter link tables. For each histone mark and genomic context (promoter or enhancer), SJSA1 and osteoblast gene sets were assembled. Then, epi-PCGs were defined as protein-coding genes present in SJSA1 but absent in osteoblast across the 12 mark x context combinations. To focus on transcriptionally relevant loci, the epi-PCG reference was intersected with TARGET-OS vs GTEx skeletal muscle differentially expressed genes (FDR < 0.05). Public GENCODE v32 gene-feature tables (transcript and exon counts and lengths) were used for ancillary comparisons of gene-structure properties between epi-PCGs and non-epi-PCGs.

Signature scoring, clustering, and survival of epigenetic scores

For each sample, single-sample GSEA (ssGSEA) enrichment scores were computed for 12 histone-mark signatures (six modifications in both promoter and enhancer), restricted to the epi-PCG universe. In parallel, pathway activity was quantified using public MSigDB collections to provide orthogonal biological context: Hallmark (H). Molecular subtypes were identified by consensus clustering of the epi-PCG ssGSEA matrix using ConsensusClusterPlus (maximum K = 10, 1000 resamples, pItem = 0.8, partitioning around medoids, Spearman distance). Cluster number was selected from the consensus CDF and delta-area plots together with inspection of consensus heatmaps, yielding a stable K = 3 solution (clusters C1 to C3). Overall survival was compared across clusters by Kaplan-Meier analysis with log-rank testing (survival::survdiff). Hazard ratios with 95% confidence intervals were derived from observed and expected event counts in the log-rank table and visualized with survminer::ggsurvplot. Histone mark signatures identified for H3K27ac_enhancer were also applied to TCGA-SARC samples.

Pathway association and immune infiltration analyses of epigenetic scores

To relate histone-mark activity to coordinated biology, Spearman correlations were computed between epi-PCG ssGSEA scores and MSigDB Hallmark module scores and summarized results as correlation heatmaps and bubble plots (color for effect size, size for P-value strata). Immune infiltration was profiled from bulk RNA-seq using CIBERSORT to infer immune cell fractions. Where

relevant, group differences were tested with two-sided Wilcoxon rank-sum tests. An interferon-gamma signature was scored per sample as a proxy for cytotoxic or inflammatory activity and compared across analytic groups. Unless specified otherwise, statistical tests were two-sided. Multiple-testing correction was applied to TARGET vs GTEx differential expression (FDR < 0.05). Nominal P values were reported for exploratory correlation and group-comparison visualizations.

Histone-deacetylase analysis of TARGET-OS and TCGA-SARC

TCGA-SARC and TARGET-OS cohorts were obtained from the GDC consortium using TCGA-SARC) with TCGABiolinks R package. A total of 342 patients were included (n= 84 TARGET-OS and n=258 TCGA-SARC). Only primary tumors were included. Clinical variables, including survival status and time to event, were retrieved, as well as gene expression data (STAR-Counts) as TPM. Log2 transformed TPM values were batch corrected using sva (Combat). Output corrected files were used for subsequent analyses. Survival plots were generated using the survminer package and plotted using ggsurvplot. For HDAC1/3 survival analysis, gene expression data were extracted and merged with clinical variables. Combined expression was calculated as the geometric mean of HDAC1/3, log2-transformed, and standardized using Z-scores. Patients with a standard deviation from the mean higher than 1.25-fold were classified as “Very High Expression”, and the rest were included as “Lower expression”.

For VGLL3 and YAP1 levels, expression levels were categorized as high (≥75th percentile) or low (≤25th percentile). Patients exhibiting High YAP1/Low VGLL3 or Low YAP1/High VGLL3 profiles were assessed for survival differences using Kaplan–Meier estimation with survival and survminer R packages. Patients not meeting this criterion were excluded. Survival differences were measured using log-rank tests.

Entinostat sensitivity and cell line RNA expression

Broad Institute DepMap portal was used to investigate top correlated gene expression to entinostat sensitivity. Entinostat drug sensitivity AUC (Sanger GDSC2: 1593) for 547 different cell lines was retrieved from Cancer Cell Line Encyclopedia, together with batch-corrected expression in log2

(TPM+1) for top correlated genes, including *YAP1*, *AMOTL2*, and genes of interest in the Hippo pathway, *CTGF* and *VGLL3* (public 24Q2), and further analyzed for correlation. Soft tissue and bone sarcoma cell lines were further selected for independent evaluation in GraphPad.

Genomic amplifications in cancer and immune correlations

Assessment of genomic amplifications of VGLL3 in TCGA-SARC and comparison with CD8 T cell infiltration were performed in TIMER 2.0 [3].

Immune deconvolution and correlation with Hippo pathway genes

The immunedeconv R package was used to predict immune infiltrates from bulk RNAseq data, from which, MCPcounter algorithms were used ([4] For tissue-resident memory T cells, effector T cells, central memory, effector memory T cells, signatures were predicted following MCPcounter algorithm of log2 geometric mean of the expression of signature genes. Correlation between gene expression values and infiltration signature scores were calculated in R using Pearson correlation, and survival curves were plotted by ggsurvplot R package.

Patient-derived spheroids RNA sequencing

Bulk RNAseq of patient-derived spheroid samples was carried out at Novogene in NovaSeq X Plus Series (PE150) before passing exhaust quality control, followed by mRNA library preparation with poly A enrichment and Standard Analysis. Deseq2 package was utilized to perform differential gene expression identification and count normalization. For identification of affected pathways upon entinostat treatment in CBOS1 and MFS1 spheroids, Deseq2 normalized gene counts were used as input for GSEA analysis using the KEGG Legacy Pathways from MSigDB using the NovoMagic platform. KEGG orthology categories with more significant hits were selected and plotted using ggplot2. GSEA plot for Hippo pathway was reproduced using replotGSEA Rtoolbox package.

The main Hippo pathway genes were classified as Hippo on or off based on their principal role in the pathway. Consequently, differential gene expression analysis (DEG) from the Novogene platform was used as input to generate bubble plots with ggplot in R Software.

For knock-down analysis, output from differential gene expression from Deseq2 from MFS1 patient corresponding to: shSCR vs shYAP1, shSCR vs shVGLL3 and, MFS1 DMSO vs MFS1 entinostat. Differentially expressed genes with a p adjusted value <0.05 and a log2FC of +- 0.53 (corresponding to 1.5-fold change), were selected. Up and downregulated genes were separated per each comparison. Venn diagrams were generated using Interactivenn [5], and intersected genes were used as input for pathway enrichment analysis using enrichR package and plotted using ggplot2.

Immunotherapy response analysis

For the analysis of STS patients undergoing ICI therapy, (GSE213065) [6], bulk RNA-seq normalized TPM and clinical data were downloaded from the Gene Expression Omnibus (GEO). Patients who received immunotherapy (Nivolumab/Ipilimumab) as well as Nivolumab/Ipilimumab/Cryotherapy were selected. For patients presenting with longitudinal lesions (T1, T2, and T3), the lesion closest to the start of ICI treatment was selected, provided the patient had not received any other therapy between lesions. For the remaining patients, T1 was selected. The average gene expression value was calculated when patients had sequenced two different areas of the same lesion, to account for tumor heterogeneity in the same tumor. Spearman correlation analyses were performed to examine the relationship between the frequency of tumor regression or progression and other variables. The two signatures, consisting of VGLL3 and ITGAE, or HDAC1, HDAC3, YAP1, and AMOTL2, were generated by the means of log2(TPM+1) of each gene in the signature. Survival analysis was performed by dividing patients into two groups based on the median value. Log-rank test was performed to test survival differences, along with Cox model for hazard ratio evaluation.

Single-cell data analysis

Patient synovial sarcoma samples from GSE131309[7] were analyzed in the Broad Institute Single Cell platform (https://singlecell.broadinstitute.org/single_cell)

Statistics

Statistical analysis was performed using GraphPad Prism 10. Data are presented as mean ± standard deviation (SD). For two-group comparisons, Student's t-test was used for parametric data, and the Mann-Whitney U test for non-normal data. One-way ANOVA was applied for multiple group comparisons. Two-way ANOVA was used for experiments with two independent variables. In R software, Kaplan–Meier survival curves were generated using the survival package. Differences between groups were assessed using two-sided log-rank tests. Cox proportional hazards models were applied to estimate hazard ratios (HRs) and 95% confidence intervals (CIs) where appropriate. Sample sizes (n) correspond to the number of cases with available survival information and the relevant stratification variable. For analyses involving multiple pairwise group comparisons, p-values were adjusted using the Benjamini–Hochberg (BH) procedure. Correlations were assessed using Pearson's or Spearman's correlation coefficients. Normality was tested using the Shapiro-Wilk test. Single-sample gene set enrichment scores (ssGSEA) were computed using ssGSEA (GSVA framework) from normalized expression matrices and predefined gene sets. Group comparisons were performed using the indicated non-parametric tests. When evaluating multiple gene sets and/or multiple immune-infiltration metrics, p-values were corrected for multiple testing using the Benjamini–Hochberg false discovery rate (BH-FDR) procedure, unless otherwise specified. Sample sizes (n) reflect cases with complete data for each analysis. The number of replicates, significance levels, and statistics used are detailed in the figure legends.

Supplementary Materials Table

Antibodies

Specificity	Dilution	Conjugated	Reference
h/m-H3K27ac	1:100	AF488	Cell signaling (D5E4)
h-CD4	1:100	SB436	Invitrogen (62-0049-42)
h-CD3	1:100	BV650	BD Horizon (563852)
h-CD8	1:100	Pe-Cy7	Invitrogen (25-0088-42)
h-CD103	1:50	APC	BioLegend (350215)
i-A/i-E	1:100	PerCP	BioLegend (107623)
H-2k(d)	1:100	PE	BD (553566)
m-CD45	1:100	AF700	BioLegend (103128)
m-PD-L1	1:100	APC	BD Pharmingen (564715)
m-CD11b	1:100	Pe-Cy7	eBioscience (25-0112-82)
m-Ly6G	1:100	V450	BD (560603)
m-Ly6C	1:100	AF647	BioLegend (128010)
m-CD11c	1:100	PE	eBioscience (11-0114-82)
m-F4-80	1:100	BV605	BD (565612)
m-CD86	1:100	PerCP/Cy5.5	BioLegend (105028)
m-CD103	1:100	BV421	BioLegend (121421)
m-CD3e	1:100	BV650	BD (564378)
m-CD44	1:100	FITC	BD (553133)
m-CD4	1:100	PerCP	BD (553052)
m-CD127	1:100	PE	BD (552543)
m-Foxp3	1:100	PE-CF594	BD Horizon (562466)
m-CD62L	1:100	APC	BioLegend (104411)
m-CD8a	1:100	APC-Cy7	BioLegend (100713)
m-GZMB	1:100	PB	BioLegend (515405)
m-IFNγ	1:100	BV785	BioLegend (505837)
m-TNFα	1:100	APC	BioLegend (506308)
h-Granzyme B	1:40	FITC	Biolegend (515403)
h-CD45RA	1:100	PE	Biolegend (304108)
h-CD69	1:50	AF700	BD (560739)
mAb IgG Isotype control	1:200	AF488	Cell signaling (2975S)
h-CD45	1:100	PerCP-eFluor710	Invitrogen (46-0459-42)
h-CCR7	1:50	BV785	Biolegend (353230)

Table 1. List of flow cytometry antibodies

Immune population	Markers
In vivo phenotyping of Immune populations	
CD4 T cell	CD45+, CD3+, CD4+CD8-
CD8 T cell	CD45+, CD3+, CD4-CD8+
Regulatory T cell	CD45+, CD3+, CD4+CD8-, FoxP3+
Central memory T cell (Gated on different subsets)	CD44+, CD62L+
(Gated on different subsets)Naive T cell	CD44-, CD62L+
(Gated on different subsets) Effector/memory T cell	CD44+, CD62L-
CD8 Tissue-resident memory	CD45+, CD3+, CD8+CD4-, CD103+
Neutrophils	CD45+, CD11b+, Ly6G+
Dendritic cells (DC)	CD45+, Ly6G-, F4-80-, CD11c+, i-A/i-E+
Plasmacytoid DC-like (pDC-like)	CD45+, Ly6G-, F4-80-, CD11c+, i-A/i-E+, CD11b-, Ly6C+
Conventional DC Type 1-like (cDC1-like)	Singlets SSC-H/SSC-A, Live, CD45+, Ly6G-, F4-80-, CD11c+, i-A/i-E+, CD11b-, Ly6C-
Conventional DC Type 2-like (cDC2-like)	CD45+, Ly6G-, F4-80-, CD11c+, i-A/i-E+, CD11b+, Ly6C-
Monocytes	CD45+, Ly6G-, F4/80-, CD11c-, CD11b+, Ly6C+
General Macrophages	CD45+, Ly6G-, F4-80+
Alveolar macrophages (AM)	CD45+, Ly6G-, F4-80+, CD11b-, CD11c+
Interstitial Macrophages (IM)	CD45+, Ly6G-, F4-80+, CD11b+
IM1-like	CD45+, Ly6G-, F4-80+, CD11b+, iAiE-, CD11c-
IM2-like	CD45+, Ly6G-, F4-80+, CD11b+, iAiE+, CD11c-
IM3-like	CD45+, Ly6G-, F4-80+, CD11b+, iAiE+, CD11c+
In vitro phenotyping	
CD4 T cells	CD3+, CD8-CD4+
CD8 T cells	CD3+, CD8+CD4-
Central memory CD8 T cell	CD3+, CD8+CD4-, CCR7+, CD45RA-
Effector/memory CD8 T cell	CD3+, CD8+CD4-, CCR7-, CD45RA-
Terminally differentiated effector CD8 T cell	CD3+, CD8+CD4-, CCR7-, CD45RA+
Tissue-resident memory CD8 T cell	CD3+, CD8+CD4-, CD103+ or CD103+/CD69+

Table 2. Markers utilized for immune cell characterization in flow cytometry

Oligonucleotides (qPCR primers for indicated genes)

Reagent	Source	Forward	Reverse
ACTB	Integrated DNA technologies	GCACTCTTCCAGCCTTCCTT	CTCCTTCTGCATCCTGTCGG
VGLL3	Integrated DNA technologies	CCAACTACAGTCACCTCTGCTAC	ACCACGGTGATTCCTTACTCTTG
YAP1	Integrated DNA technologies	TGTCCCAGATGAACGTCACAGC	TGGTGGCTGTTTCACTGGAGCA

Table 3. List of qPCR primers

Methods references

1. Wickström S, Lövgren T. Immune Checkpoint Blockade, Methods and Protocols. Methods Mol Biol. 2019;1913:105–18.

2. Santos MCD los, Lundqvist A. Evaluation of lymphocyte infiltration into cancer spheroids by immunofluorescent staining and 3D imaging. Methods Cell Biol. 2024;191:269–87.

3. Li T, Fu J, Zeng Z, Cohen D, Li J, Chen Q, et al. TIMER2.0 for analysis of tumor-infiltrating immune cells. Nucleic Acids Res. 2020;48:W509–14.

4. Becht E, Giraldo NA, Lacroix L, Buttard B, Elarouci N, Petitprez F, et al. Estimating the population abundance of tissue-infiltrating immune and stromal cell populations using gene expression. Genome Biol. 2016;17:218.

5. Heberle H, Meirelles GV, Silva FR da, Telles GP, Minghim R. InteractiVenn: a web-based tool for the analysis of sets through Venn diagrams. BMC Bioinform. 2015;16:169.

6. Subramanian A, Nemat-Gorgani N, Ellis-Caleo TJ, IJzendoorn DGP van, Sears TJ, Somani A, et al. Sarcoma microenvironment cell states and ecosystems are associated with prognosis and predict response to immunotherapy. Nat Cancer. 2024;5:642–58.

7. Jerby-Arnon L, Neftel C, Shore ME, Weisman HR, Mathewson ND, McBride MJ, et al. Opposing immune and genetic mechanisms shape oncogenic programs in synovial sarcoma. Nat Med. 2021;27:289–300.
